# Urine to highly porous heteroatom-doped carbons for supercapacitor: A value added journey for human waste

**DOI:** 10.1038/s41598-017-11229-6

**Published:** 2017-09-07

**Authors:** Fatemeh Razmjooei, Kiranpal Singh, Tong Hyun Kang, Nitin Chaudhari, Jinliang Yuan, Jong-Sung Yu

**Affiliations:** 1 Department of Energy Systems Engineering, DGIST, Daegu, 42988 Republic of Korea; 2 Department of Chemistry, Korea University, Seoul, 02841 Republic of Korea; 3 Faculty of Maritime and Transportation, Ningbo University, Ningbo, 315211 China

## Abstract

Obtaining functionalized carbonaceous materials, with well-developed pores and doped heteroatoms, from waste precursors using environmentally friendly processes has always been of great interest. Herein, a simple template-free approach is devised to obtain porous and heteroatom-doped carbon, by using the most abundant human waste, “urine”. Removal of inherent mineral salts from the urine carbon (URC) makes it to possess large quantity of pores. Synergetic effect of the heteroatom doping and surface properties of the URC is exploited by carrying out energy storage application for the first time. Suitable heteroatom content and porous structure can enhance the pseudo-capacitance and electric double layer capacitance, eventually generating superior capacitance from the URC. The optimal carbon electrode obtained particularly at 900 °C (URC-900) possesses high BET surface area (1040.5 m^2^g^−1^), good conductivity, and efficient heteroatom doping of N, S, and P, illustrating high specific capacitance of 166 Fg^−1^ at 0.5 Ag^−1^ for three-electrode system in inorganic electrolyte. Moreover, the URC-900 delivers outstanding cycling stability with only 1.7% capacitance decay over 5,000 cycles at 5 Ag^−1^. Present work suggests an economical approach based on easily available raw waste material, which can be utilized for large-scale production of new age multi-functional carbon nanomaterials for various energy applications.

## Introduction

The unique characteristics of carbon allow a versatility unlike other elements, which make it possible for carbon atom to bond readily with neighboring atoms with different hybridizations, bringing about allotropes of completely different properties^1–5^. The control over manipulation of carbon’s physicochemical properties, such as electrical conductivity, chemical inertness along with tunable electrical and thermal properties, has given carbon an immediate application in various aspects of electrochemical energy conversion and storage^6–16^. Among all carbon materials, novel porous carbon materials owing to their large surface area with well-made pore size distribution, and relatively good electrical property are being persistently developed and used as a trusted electrode material in wide variety of advanced energy applications such as batteries, supercapacitors, and fuel cells^17–27^.

Contingent on mechanism of charge storage, the capacitance value of supercapacitors is determined mainly by two storage principles^28–30^. This can be categorized in electrochemical double layer capacitance (EDLC) arising from the non-faradaic charge separation at an electrode/electrolyte interface and pseudo-capacitance originating from fast sequence reversible faradaic redox reactions of electroactive species. Typically, if two types of capacitance of a supercapacitor, EDLC and pseudo-capacitance, work together, overall capacitance of a supercapacitor drastically increases. Generally, carbon-based active materials with high surface area are utilized in the former category, whereas conducting polymers and transition metal oxides/hydroxides are used in the latter one^10, 28, 31–35^. Recently, it is reported that certain heteroatom functionalities due to their electron donor or electron acceptor properties, when doped into the carbon, can improve the surface wettability of electrode through enhanced number of hydrophilic polar sites on the electrode surface^36, 37^. This could be due to the fast faradic redox reaction between the ions in the electrolyte and electrode, which can enhance capacity and energy density while maintaining power density^36, 37^. Therefore, high specific surface area carbon materials functionalized with a well-distributed micro-/mesopores and with certain amounts of heteroatoms can be served as an electrode material for supercapacitor, which can possibly deliver high capacitance with high energy density and power density.

The development of relatively simple and cost-effective strategy to synthesize carbon-based materials that have excellent structural and functional properties is highly sought and challenging for energy storage applications. Among the various synthesis pathways for the fabrication of porous carbon materials, hard or soft sacrificial template by using silica or surfactant, pyrolysis of polymer blends, aerogel, and pyrolysis of heteroatom-enriched biomass followed by various activation methods have attracted considerable attention^38–42^. In spite of their ability to form large surface area, favorable pore-size distribution, and rather electrically conductive carbon materials, most of these synthetic methods are very complex due to the use of either environmentally harmful activation or time-consuming templating process requiring stringent reaction conditions^38–42^. On the other hand, incorporation of heteroatom into the porous carbon network has received much attention in electrochemical energy conversion and storage devices^25, 43–46^. Methodologies to obtain a heteroatom-doped porous carbon using more convenient way have been attracting huge research interests over the past decade. In this regard, biomass residues derived from plants and animals have been considered as an excellent candidate for generating heteroatom-doped porous carbon framework due to the presence of large amounts of organic and inorganic compounds in their backbones^43, 44, 46^.

Recently, we have discovered that abundantly available human organic waste “urine” can be an excellent candidate for the oxygen reduction reaction catalyst, as it can be easily converted to the highly efficient heteroatom-doped porous carbon material (hereafter URC)^47^. Apart from water (95% of urine), urea or carbamide is the main component of urine by weight, which is one of the most major precursors for nitrogen. Besides that, the other remaining constituents of urine are chloride, sodium, potassium, silica, sulfur, creatinine (also nitrogen source), other dissolved ions, and inorganic and organic compounds^47^. Therefore, presence of high amount of heteroatoms and metals in the form of salts, which can act as active sites and progen, respectively, can make urine as an auspicious candidate to develop a new family of heteroatom-doped porous carbon materials for high performance supercapacitor.

Another perspective is that converting a urine disposal to suitable alternative source of energy materials can also prevent the water pollution. Some bacteria in water can oxidize nitrogen and phosphorous present in urine to nitrates and phosphates. During this oxidation process, a large quantity of oxygen dissolved in water needed for aquatic animals is consumed. Moreover, diseases (like dysentery, typhoid, cholera, etc.) can be conveyed from urine, which are transmitted through water supplies. Considering the above mentioned facts, heteroatom-doped porous carbon materials obtained from urine (URC) material not only can be environmentally and economically appealing but also can be applied in many forms of energy related devices.

In this study, for the first time, we have investigated the potential applicability of URC material as an electrode for supercapacitor. Present work enables a large-scale production of conductive heteroatom-doped porous carbon by using a convenient template-free approach, consisting of urine drying and then pyrolysis at different temperatures followed by acid-treatment causing to create various porosity in carbon matrix. Interestingly, it is found that inherent salts that are present in the urine backbone can evaporate during carbonization and the rest can be etched out from the resulting carbon during acid-treatment to create high amount of micro-/mesopores in the carbon framework. Therefore, use of urine as a carbon precursor unnecessitates the time-consuming extra activation processes with harmful activating agents and template preparation. On the other hand, the conductivity of the carbon obtained from urine, which has important effect on the overall electrochemical performance, can be controlled by regulating carbonization temperature. Therefore, in this study we have tried to optimize the carbonization temperature to highlight the contribution of surface area, conductivity, and types of heteroatoms introduced onto the carbon surface to supercapacitor performance of the URC. Consequently, the technique developed here can simultaneously optimize multiple aspects of properties of carbon, which has the potential to be used as highly efficient electrodes for supercapacitor and other electrochemical processes.

## Results and Discussion

**Figure 1:**
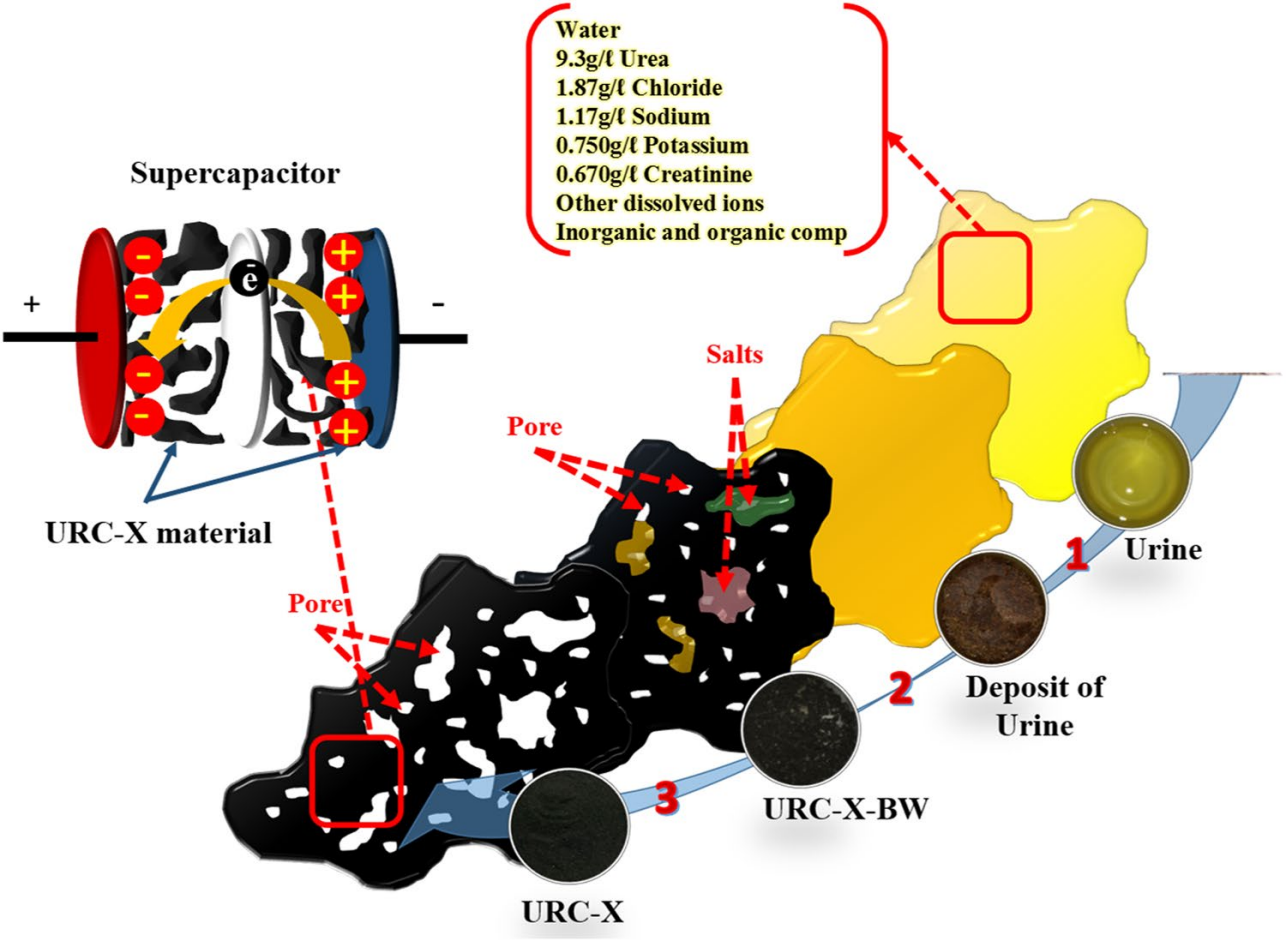
Schematic illustration of porous URC synthesis process from human urine. Step 1: a dried yellowish-brown deposit of urine obtained at 80 °C. Step 2: pyrolysis of the obtained deposit under N_2_ atmosphere. Step 3: acid-treatment to remove the mineral salts mixture to form the carbon matrix.

Overall synthetic procedure for the preparation of URC materials at different temperatures is shown in Fig. 1. Briefly, in the first step, the urine sample of a healthy individual was collected over a twenty-four hour period followed by drying at 80 °C in oven. In the second step, the obtained yellowish-brown urine deposit was carbonized at different temperatures 800, 900, and 1000 °C followed by acid washing of as-prepared carbon materials at the third step to obtain the final product URC-X, where X signs carbonization temperature (for detailed experimental procedure, please see experimental section). In general, 300–400 mg of porous URC was obtained using 1 L of human urine using such a simple template-free process. The amount of urine our body produces in a day is directly related to individual health. The normal urine output for an adult is between 1 to 2 L per day depending on health and age^47^. Therefore, from every healthy human’s body, 300–800 mg of URC can be obtained from the sterile liquid wastes per day^47^.

**Figure 2:**
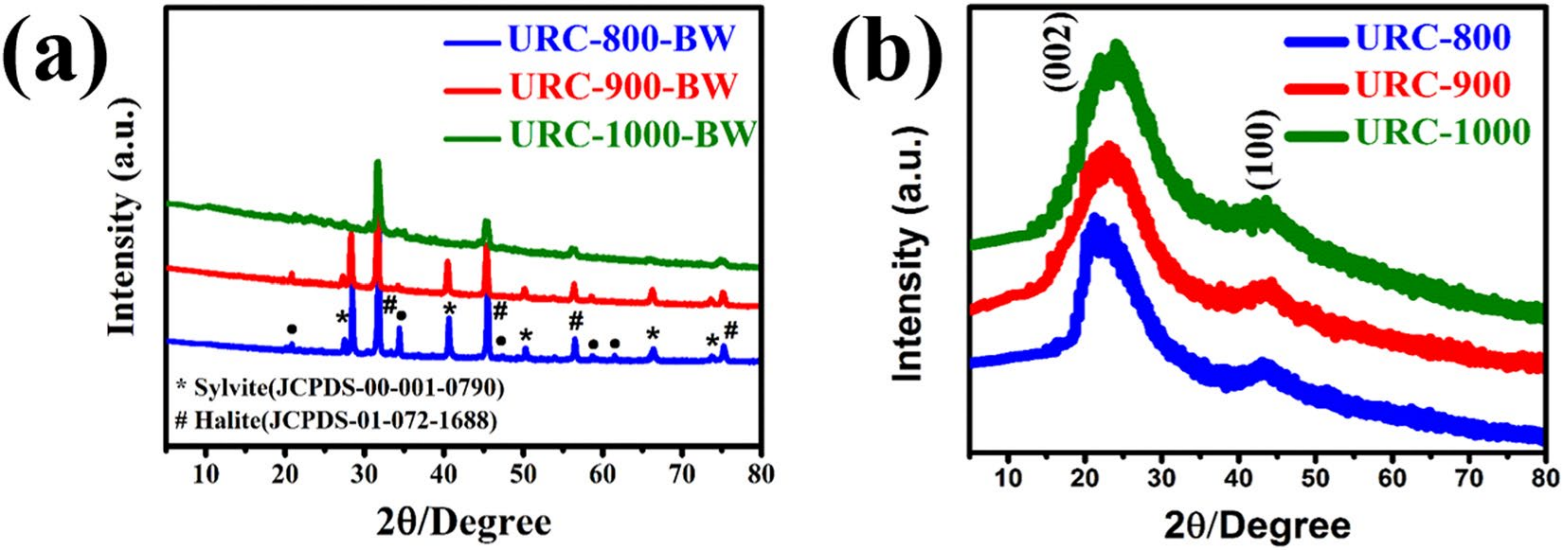
XRD patterns for the URC samples obtained from human urine at various temperatures, (**a**) before and (**b**) after acid-treatment.

To investigate the effect of pyrolysis on the crystal structure of all the obtained samples, X-ray diffraction (XRD) analysis was carried out. As shown in Fig. 2a, all the carbonized samples before acid-treatment show prominent salt peaks, mainly for sylvite (KCl, JCPDS-00–001–0790) and halite (NaCl, JCPDS-01-072-1688)^47^, with some extra impurity peaks marked with a (•) symbol. It is clearly observed that the intensity of salt peaks is much prominent for samples carbonized at lower temperature. Due to high intensity of salt peaks for samples carbonized at lower temperature signal for carbon peak at 25° is quite suppressed. However, as carbonization temperature increases, the intensity of salt peaks gradually deceases and a weak and broader diffraction peak at 25° for carbon can be evidently identified. In our previous study, it was found that sylvite salts clung to the inner wall of the furnace as a uniform layer of powder during cooling process, due to gasification of sylvite salts at high temperature, which justifies the decrement in the salt peak intensity at higher temperature^47^. XRD patterns in Fig. 2b show that all the salt peaks completely disappear after acid-treatment for URC carbon, and only two major peaks, (002) peak at ~25° and (100) peak at ~44° remained, which are characteristic of turbostratic carbon. The (002) peak intensity corresponding to coherent and parallel stacking of the graphene like-sheets increases as temperature increases, suggesting the increase of the graphitic stacking order of the carbon matrix. Furthermore, as can be seen in Fig. 2b, URC-800 prepared at relatively lower temperature of 800 °C shows the (002) peak shifted towards the lower angle as compared with that of URC-900 and URC-1000, which implies that URC-800 possesses larger interlayer spacing between graphene sheets compared with URC-900 and URC-1000^45^. This means that the samples carbonized at higher temperature, URC-900 and URC-1000 possess more intact graphitic structure.

**Figure 3:**
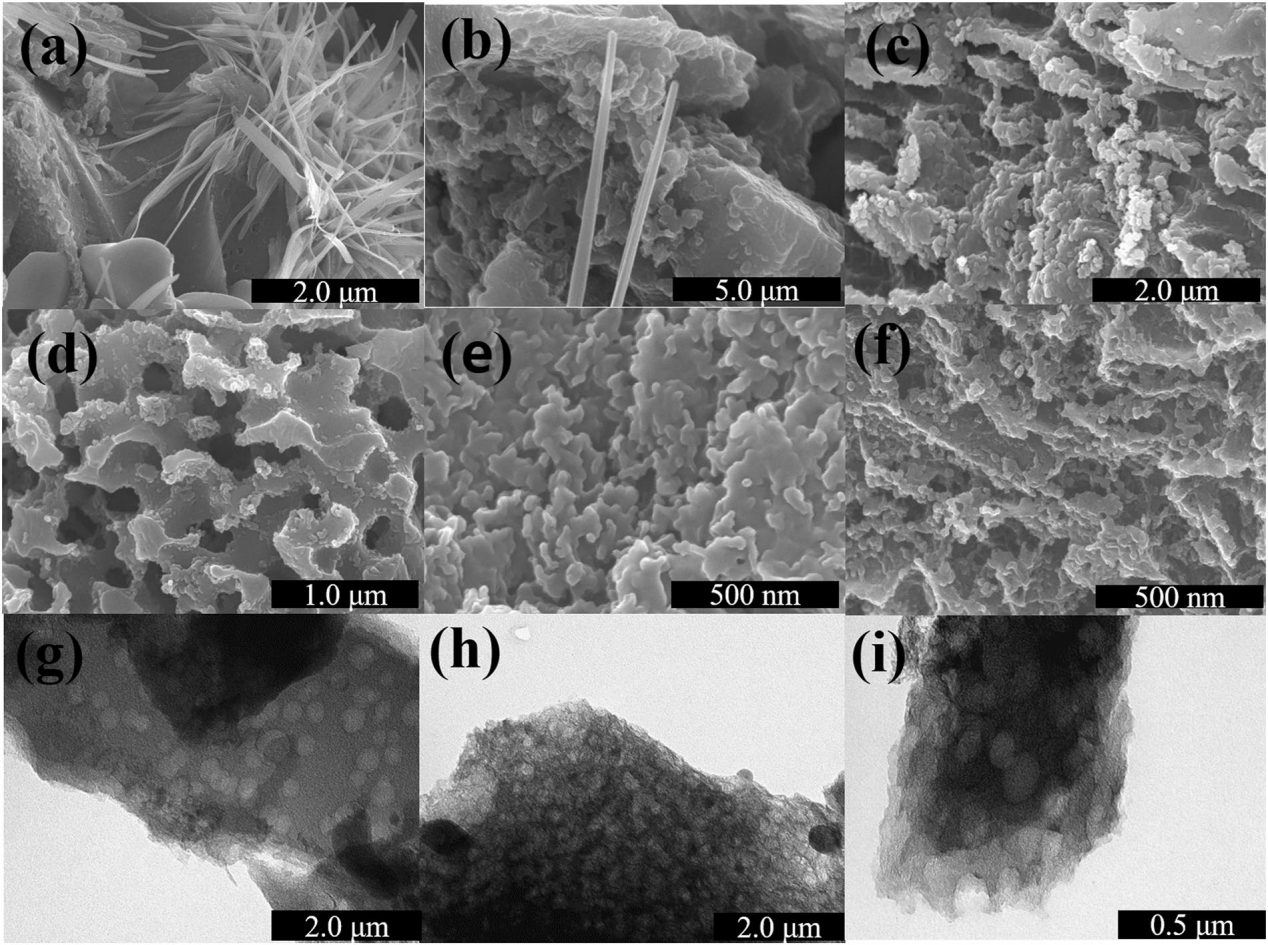
SEM images of (**a**) URC-800-BW, (**b**) URC-900-BW, (**c**) URC-1000-BW, (**d**) URC-800, (**e**) URC-900, (f) URC-1000, and TEM images of (**g**) URC-800, (**h**) URC-900, and (**i**) URC-1000.

In agreement with the XRD analysis, scanning electron microscopy (SEM) images also show the mixture of small interconnected micro-size carbon particles and rod-like rock salts for URC-800-BW and URC-900-BW as shown in Fig. 3a and b. However, upon further increasing temperature to 1000 °C, inorganic salts present in urine backbone start evaporating, and therefore almost all the fiber and niddle-like salts perish and mostly carbon material is left behind, Fig. 3c. However, as shown in SEM and transmission electron microscopy (TEM) in Fig. 3(d–i), after acid-treatment, formation of pore-like structure is clearly observed, which can be attributed to the removal of trapped mineral salts and inorganic impurities from the carbon lattice. Therefore, evidently it can be claimed that one of the main advantage of this simple synthesis approach is that without use of any activation agent, large-scale production of porous heteroatom-doped carbon is achieved by using the self-pore generating properties of inherent salts present in dried urine structure. The urine deposit contains high amount of organic compounds and mineral salts. As comparison, the SEM image of dried yellowish-brown deposit of urine obtained at 80 °C is shown in Fig. S1 of Supplementary information (SI), which consists of organic compounds and dense mixture of various salts with particle shape showing the presence of high amount of salts in the urine.

**Figure 4:**
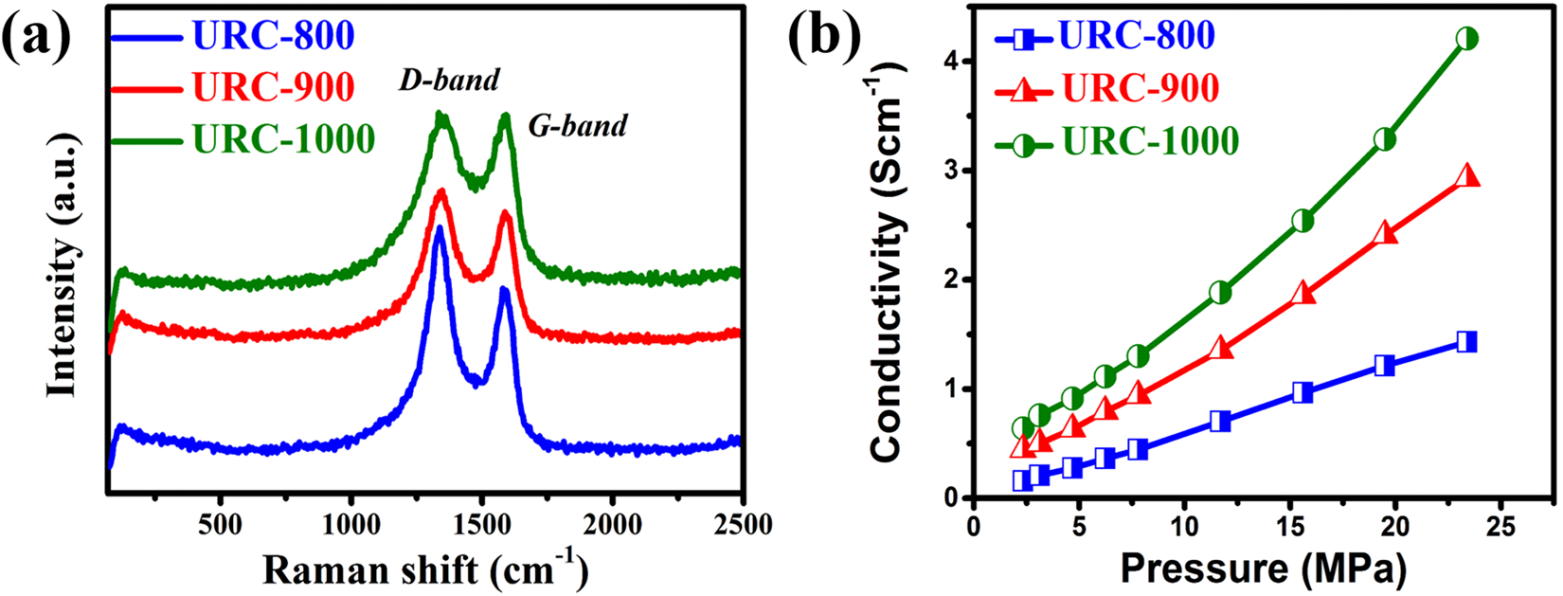
(**a**) Raman spectra and (**b**) conductivity vs. pressure curves of the URC materials obtained at various carbonization temperatures.

Raman results show two distinctive peaks at 1340 cm^−1^ and 1575 cm^−1^ designated as D and G band, respectively, for all the URC samples^48^. As can be seen in Fig. 4a, I_D_/I_G_ ratio, which is used to measure the level of disorder in graphitic structure, decreases with increasing temperature, suggesting the increase of the graphitic order of the carbon matrix. This can be deduced from the difference in I_D_/I_G_ values of URC-800 (1.25), URC-900 (1.11), and URC-1000 (0.98). This is supported by the XRD results in Fig. 2b, which indicate the increase of the graphitic stacking order of the carbon matrix as temperature increases.

Conductivity and surface area are the properties, which need a special attention while defining the electrochemical energy storage of the carbon materials. The electrical conductivity of all samples was evaluated by home-made four probe appliance as a function of pressure and a diagram of the measurement cell is shown in Fig. S2 of SI. The conductivity is found to be proportional to the applied pressure and the carbonization temperature as shown in Fig. 4b. In general, it was found that as the pyrolysis temperature was increased, the total resistivity of samples decreased^1, 25^.

**Figure 5:**
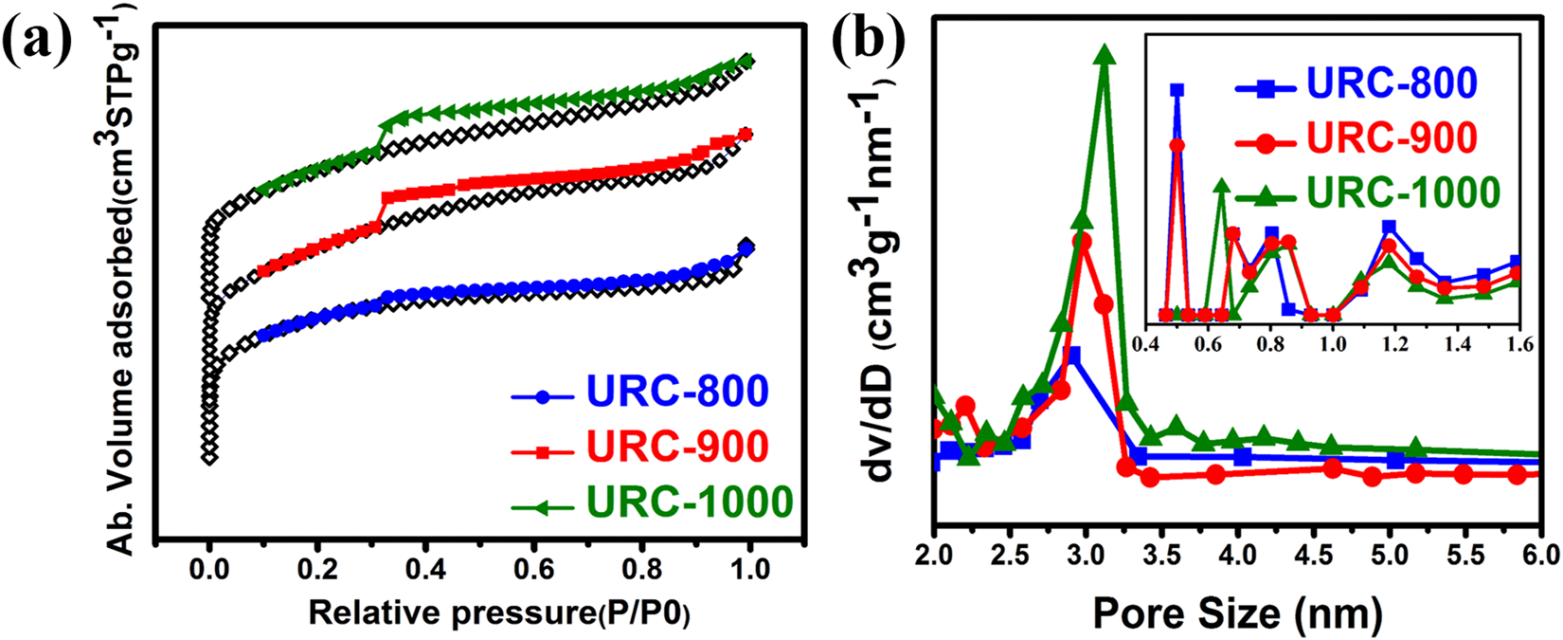
(**a**) Nitrogen adsorption-desorption isotherms and (**b**) the corresponding pore size distribution curves of all the URC samples obtained from BJH method (Inset: corresponding pore size distribution curves of all the URC samples obtained from H-K method).

As high surface area of carbon electrodes is believed to require for the permeability of electrolyte ions through the pores^1, 25^, the textural properties of all the URC materials were studied by nitrogen adsorption/desorption isothermal measurements as shown in Fig. 5. Figure 5a shows that the entire URC samples illustrate a type IV curve with noticeable hysteresis loop in the isotherms, attributed to the mesoporous materials. However, a line parallel to Y axis at lower relative pressure P/P0 (∼0.01) is obtained for all the samples, displaying good evidence for the presence of micropores as well^24^. All the parameters collected by nitrogen sorption data were summarized in Table S1 of SI. As shown in Table S1 of SI, with increasing temperature, the Brunauer-Emmett-Teller (BET) surface area decreases from 1330.6 m^2^g^−1^ for URC-800 to 1040.5 and 809.3 m^2^g^−1^ for URC-900, and URC-1000, respectively. Interestingly, while the total surface area, total pore volume, and micropore volume of each sample decrease by increasing temperature (Table S1 of SI), the corresponding mesopore volume increases with increasing temperature, which can be implied from clearly appeared hysteresis loop profile and enhanced adsorption curve at higher pressure. Formation of larger micropore volume in carbon obtained at lower temperature can be attributed to the salt removal by acid-treatment, whereas the presence of higher mesopore volume in sample obtained at higher temperature can be ascribed to the evaporation of mineral salts during high temperature carbonization^47^.

To further analyze the pore structures of URC samples, Pore-size distribution (PSD) curves were obtained using Barrett-Joyner-Halenda (BJH) model as shown in Fig. 5b. Based on the BJH model, the PSD curves exhibit that the mesopore diameter increases from 2.8 nm for URC-800 to 3.1 nm for URC-1000 by increasing temperature as shown in Table S1 of SI. A significant increase in mesopores volume and a decrease in micropore volume are also observed upon increasing temperature, which is consistent with the increase of mesopore intensity. Based on the Horvath-Kawazoe (H-K) model, the PSD curves, added in inset of Fig. 5b, also display the presence of micropores in the range of 0.5 to 1.3 nm for the obtained samples. The micropores can play a crucial role for developing the electrical double-layer surfaces to reach high capacitance, while mesopores can provide not only good charge propagation during high current loads with low resistance, but also wide transport paths for ion diffusion into micropores to enhance the capacitor performance. Therefore, presence of appropriate amount of both micro and mesopores would be beneficial for ion diffusion in supercapacitor^49–54^. Fig. S3 of SI shows the High resolution-TEM (HR-TEM) images of URCs with different magnifications that further highlight the meso and microporous structure. The bright sections are considered as pores. The mesopores of the URCs are clearly observed in Fig. S3(a–c) of SI, and the graphitic structure and microporous carbon are clearly observed in HR-TEM images in Fig. S3(d–f) of SI, which are in good agreement with BET surface area.

**Figure 6:**
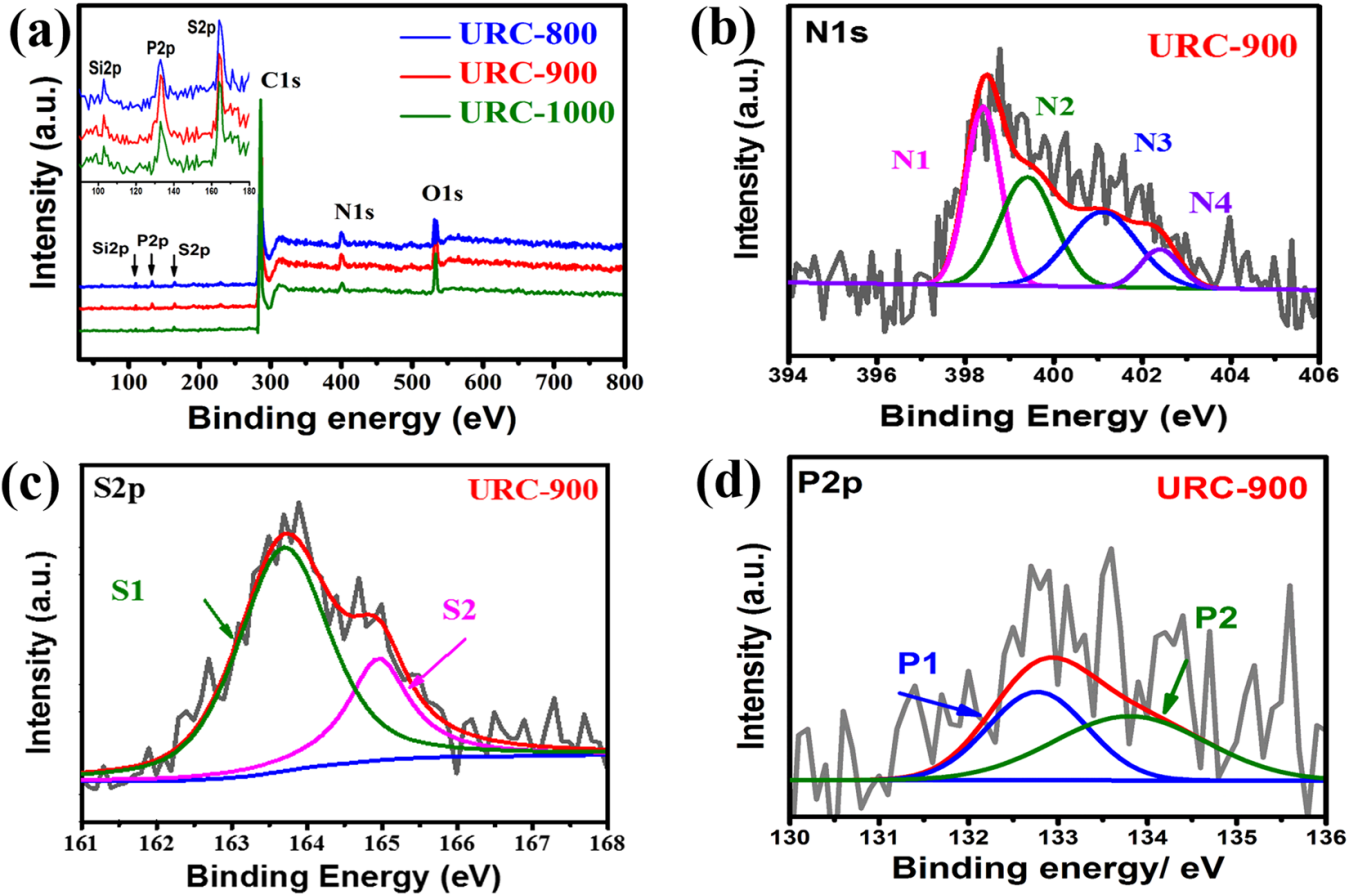
(**a**) XPS survey spectra of all the URC samples prepared at various carbonization temperatures (Inset: magnified XPS survey spectra for Si, P, and S). Deconvoluted XPS spectra of (**b**) N 1s, (**c**) S 2p, and (**d**) P 2p for URC-900.

To determine the atomic composition and the bonding state of heteroatoms in the resulting URC materials, which changes as a function of temperature growth, X-ray photoelectron spectroscopy (XPS) analysis was carried out. As can be seen in Fig. 6a, the full-range XPS survey spectra show the presence of C, O, and N as the main components of the URC materials along with the minor presence of S, Si and P peaks. Recently, it has been found both experimentally and theoretically that heteroatom such as N, S, and P can introduce defects into the carbon backbone that facilitate electrolyte penetration and ion transport, which significantly boost capacity and energy density of supercapacitors^55–57^. Among these heteroatoms, particularly N plays an important role to induce negative charge on carbon surface, improving the wettability of the interface between the electrolyte and the electrode, and to improve charge mobility on the carbon, inducing pseudo-capacitance, eventually increasing the overall capacitive performance^55, 58, 59^. Since it is known that the presence of heteroatoms at edge sites can drastically enhance the capacitive performance, P doping, which generally takes place at the edge-plane sites of the graphitic framework due to the larger atomic size (0.106 nm vs. 0.077 nm for C), can result in enhancement of the energy storage^57, 60, 61^. Besides N and P, S incorporated into the carbon materials has been also attracting a great research interest for supercapacitor since last few years^62–64^. The enhanced performance of S-doped carbon materials is attributed to the electron-rich aromatic sulphide, which causes the formation of more polarized surface under an applied electric field, facilitating charge transfer process through enhancing the dielectric constant^62^.

The total surface heteroatom contents were found to be decreased by increasing the carbonization temperature 800 to 1000 °C as expected, showing prominent changes as a function of carbonization temperature as displayed in Table 1. As nitrogen states can affect the electrochemical behavior of electrode, high resolution XPS scan displaying the effect of temperature growth on the nitrogen binding state is studied. As shown in Fig. 6b and Fig. S4 of SI, nitrogen bonding configurations within the carbon lattice can be divided into four different species according to their binding energies including N1: pyridinic-N (398.4 eV), N2: pyrrolic-N (399.4 eV), N3: quaternary-N (401.1 eV), and N4: oxides of nitrogen (402.4 eV). Specifically, with increase in the carbonization temperature, the percentage of pyrrolic and pyridinic-N starts deteriorating from URC-800 to URC-1000 due to the instability of these nitrogen species at high temperature, whereas quaternary-N species become prominent in the case of URC-1000. These results are in well agreement with the earlier reported works, which reveal that the doping environment depends on the carbonization temperature^25, 47^. According to earlier works^65–68^, it has been found that both pyridinic and pyrolic-N play an effective role on the capacitance enhancement. Generally, pyridinic-N and pyrrolic-N connect with two C atoms. Interestingly, the lone pair in pyrrolic-N is included in the π system, while the one in pyridinic-N is not included^65–68^. However, the quaternary-N refers to N atom substituting for a C atom in the hexagonal ring. Among these N species, pyridinic and pyrrolyic-N, which are readily accessible to electrolyte ions, allow fast diffusion of electrolyte ions, and are responsible for providing pseudo-capacitance, in turn enhancing the electrode capacitance^66, 67^. Therefore, the N-doped carbon with pyridinic and pyrrolyic-N species in majority is in favor of serving as an electrode for supercapacitors^66^. N content and its specific bonding types vary with the carbonization temperature as indicated in Fig. 6b, Table 1, and Fig. S4 of SI. Pyridinic and pyrrolic-N species are predominant in URC-800 and URC-900 with higher N content, while the quaternary-N is dominant in URC-1000 with lowest N content. This implies that N atoms can be mostly doped onto the carbon edges and defects at lower temperature, which improves the electrochemical behavior of carbon materials.

**Table 1:** Surface element contents obtained from the XPS analysis for URC samples.

Sample	Atomic Composition (%)					
	C1s	O1s	N1s	S 2p_3/2_	Si 2p	P 2p
URC-800	82.8	7.8	7.4	0.8	0.6	0.6
URC-900	85.6	7.1	5.7	0.7	0.5	0.4
URC-1000	89.8	6.5	2.7	0.6	0.2	0.2

The high resolution C 1s peak for all the samples shows a main peak located at 284.6 eV with tail towards higher binding energy (Fig. S5 of SI). C 1s spectra of all the three samples can be deconvoluted into 3 different components, which are (C1): C=C sp^2^ (~284.6 eV), (C2): sp^3^ hybridized carbon (~285.3 eV), and (C3): C-N, C-O (~286.7 eV), showing that most of the carbon atoms remain within the two-dimensional honeycomb lattice of sp^2^ bonded matrix. As shown in Table 1, the carbon content increases by increasing temperature from 82.8 for URC-800 to 85.6 and 89.8 wt% for URC-900 and URC-1000, respectively, which can be due to the decreasing of heteroatom content. On the other hand, as shown in Table 1, with increasing temperature, the total O content of URCs was decreased from 7.8 to 7.1 and 6.5 at.% for URC-800, URC-900, and URC-1000, respectively. This still validates the presence of a significant amount of oxygen-containing functional groups in the URC samples. Along this line, as shown in Fig. S5 of SI, with increase in the carbonization temperature, the percentage of C-O fraction (C3) starts deteriorating from URC-800 to URC-1000. It is reported that increase in the population of oxygen groups is beneficial for capacitance promotion, but the advantage can be subsided with decreasing conductivity due to presence of high amount of oxygen groups^1^. Therefore, only appropriate proportion of oxygen can effectively improve the capacitance. The above results clearly show that appropriate carbonization temperature is critical for preserving desirable functional groups within the carbon frameworks^69, 70^.

Furthermore, Table 1 summarizes the content and change of other minor elements (less than 1%) detected by XPS in different URC samples. As shown in Table 1, S and P content also display temperature dependence behaviour. As expected, an increase in temperature results in a decrease in S and P content. Figure 6c shows that deconvolution of S 2p for URC-900 gives two prominent peaks, (S1): S2p_3/2_ (163.9 eV) and (S2): S2p_1/2_ (165.3 eV) attributed to aromatic sulphide groups, which are responsible for providing a more polarized surface under an applied electric field, leading to increased dielectric constant and improved charge transfer process^62^. As can be seen in Fig. 6d, the P 2p spectrum is deconvoluted into two peaks for URC-900 with binding energy of 132.7 and 133.3 eV, which can be assigned for (P1): P-C and (P2): P-O in the form of C-PO_3_ (pyrophosphonate)/or tetrahedral PO_4_ (pyrophosphate), respectively. While P1 bonding reveals the incorporation of P atoms into the graphene sheets of carbon-based catalyst, P2 bonding reveals the oxygen functional groups in the carbon framework. P with a lone pair of electrons can induce Faradaic reactions in addition to EDLC, indicating an increase in specific capacitance^71, 72^. On the other hand, it is reported that chemically stable P2 (phosphate groups) species can block the unstable active oxygen sites (oxygen sites, which are not stable and can cause capacitance deterioration during the charging and discharging process)^72^, which has positive effects on the improvement of the capacitance retention ratio, and it also widens the operating potential window for higher energy density^73, 74^.

Elemental mapping is performed to study the element distribution of N, P, S, O, and Si incorporated into URCs. As shown in Fig. S6 of SI, EDX elemental mapping images indicate the existence of C and heteroatoms including N, P, S, O, and Si on the surfaces of URC-900. These foregoing results demonstrate that N, P, S, O, and Si have been successfully and uniformly incorporated into carbon framework, which would play a significant role in enhancing capacitance.

**Figure 7:**
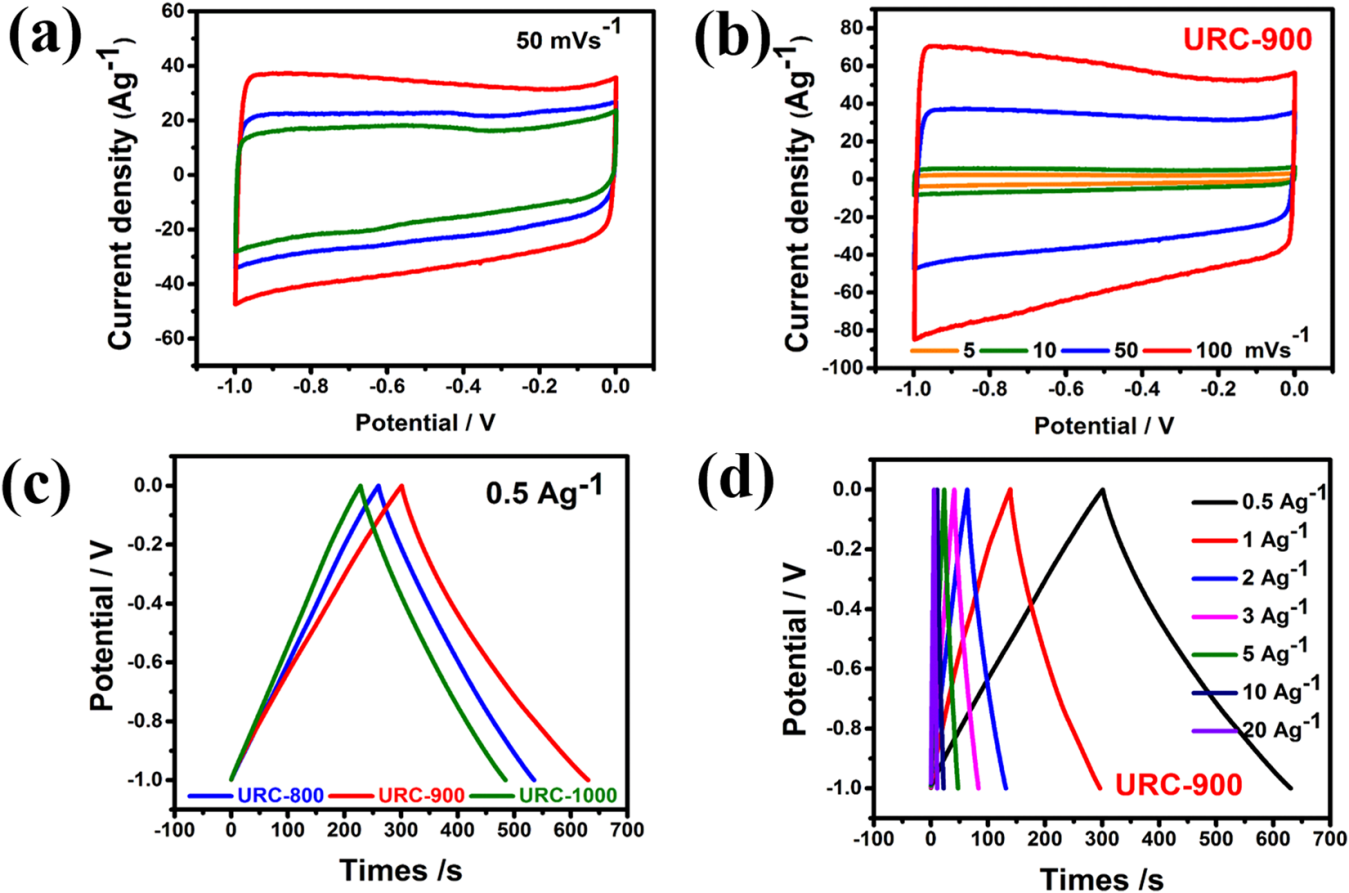
CV profiles of (**a**) all the URC materials at 50 mV s^−1^ potential scan rate and (**b**) URC-900 at different potential scan rates from 5 to 100 mVs^−1^. Galvanostatic CD profiles of (**c**) all the URCs at a constant current density of 0.5 Ag^−1^ and (**d**) URC-900 at different current densities from 0.5 to 20 Ag^−1^.

To evaluate the characteristics of the URCs as electrode materials for supercapacitor and probable effect of heteroatom doping, surface area, and conductivity on electrochemical performance, they are tested in a three-electrode configuration in 6.0 M aqueous KOH electrolyte. CV for URC electrodes was performed at a scan rate of 50 mVs^−1^, and results are displayed in Fig. 7a. All the URC electrodes retain a relatively symmetrical rectangular shape CV curves, displaying the highly capacitive nature with fast ion diffusion and good charge propagation at a medium voltage scan rate.

The area under the cyclic voltammetry (CV) curves progressively changes with variation of pyrolysis temperature, which represents the capacitance order obtained for as-prepared electrodes: URC-900 > URC-800 > URC-1000. These results indicate that URC-900 shows the highest capacitance among all the URC electrodes. As shown in Fig. 7b, as the sweep rate increases, the CV loop area for URC-900 becomes much broader, following the general trend. CV curves of the URC materials at a different scan rates are also shown in Fig. S7 of SI. As the scan rate increases, the CV curves become broader, but excellent symmetric rectangular shape of CV curves is still retained at both low and high scan rates showing facile charge movement even at higher scan rate. Additionally, a pair of anodic and cathodic peaks at the lower scan rates can be seen, which manifests the occurrence of reversible redox reactions on URC electrode probably due to the presence of active heteroatom and oxygen functionalities on the electrode surface. These results show that the capacitive response for URC materials is derived from a combination of both EDL capacitance and pseudo-capacitance. Furthermore, as shown in Fig. S8 of SI, the maximum capacitance value of 161.64 Fg^−1^ was obtained from CV curve at 5 mVs^−1^ for URC-900 compared with that of URC-800 (124.0) and URC-1000 (111.0). URC-900 also shows high capacitance retention of around 62% at high scan rate of 100 mVs^−1^, demonstrating its good rate capability at high scan rates.

To further evaluate the capacitance performance, galvanostatic charge-discharge (CD) was carried out by chronopotentiometry between 0.0 and −1.0 V at different current densities. Figure 7c shows comparative galvanostatic CD curves of all the URC materials at a constant current density of 0.5 Ag^−1^. The galvanostatic CD curves show almost isosceles triangular shapes, suggesting good columbic efficiency and typical capacitive behaviour for all the URC electrodes^37^. The obtained specific capacitances are 139, 166, and 129 F.g^−1^ for URC-800, URC-900, and URC-1000, respectively, at a constant current density of 0.5 Ag Ag^−1^ in 6.0 M KOH. The galvanostatic CD profile curves at various current densities for all the URC materials at the same voltage window are also shown in Fig. 7d and Fig. S9 of SI. The discharging time of the URC-900 is noticeably longer compared with that of other URC materials at both low and high current densities, implying its higher capacitance in URC-900, which is in well agreement with those obtained from CV performance.

The prominent electrochemical performance of URC-900 encouraged us to test its performance in two-electrode system. Similar to the results from the three-electrode measurement, URC-900 exhibits good double-layer capacitive behaviours and its CV keeps the rectangular symmetry at different scan rates between 0 and 1.2 V, even at a scan rate as high as 200 mV s^−1^ (Fig. S10a of SI), indicating fast electron and ion transport in the URC-900 electrode. As shown in Fig. S10b of SI, the galvanostatic CD curves show almost isosceles triangular shapes similar to the three-electrode system. As can be seen in Fig. S10c of SI, URC-900 shows a reasonable specific capacitance of 254.12 F g^−1^ at a current density of 0.1 A g^−1^. In practical applications, the specific energy density and power density are the most important factors determining the performance of electrochemical supercapacitors^75, 76^. The excellent performance of the URC-900 can also be gleaned from its superior energy density retention over the complete power density range. As can be seen in Fig. S10d of SI, URC-900 delivers reasonable energy density value (12.7 to 6.10 WhKg^−1^) in the range of reasonable high power density value (60.07 to 337.84 WKg^−1^) at the operating voltage window of 1.2 V, which has a direct effect on both energy density and power density.

In supercapacitor based on heteroatom-doped carbon materials, capacitance, or the ability to store an electrical charge, can be enormously influenced by several parameters; surface area, conductivity, heteroatom content, and types of heteroatom species, which require an optimization. Since the surface area, amount of heteroatom content, and conductivity varies significantly as a function of carbonization temperature, the trade-off among them should be discussed fully with the temperature for better understanding of electrochemical properties. In this present study, we observe an increase in specific capacitance of URC-900 compared with that of URC-800, which has a higher surface area and higher heteroatom content than those of URC-900. This increase can be attributed to the higher conductivity of URC-900 compared with that of URC-800. Although a higher conductivity of URC-900 can contribute towards the higher capacitance, this cannot be the only reason for capacitance increment. As adequate pore size distribution is crucial for capacitive behavior since EDLC is greatly affected by the actual interface between the electrode and electrolyte. URC-900 with lower surface area but with much more appropriate distribution of micro-/mesopores than URC-800 shows higher capacitance. On the other hand, although URC-1000 is highly conductive, it shows lower capacitance compared with URC-900, which might be due to its low surface area, low heteroatom content, and absence of sufficient amount of active heteroatom-doped species at the defects and edges. It is well known that pyridinic-N and pyrrolic-N, generated on the edge sites of the graphitic framework, can deliver significantly higher capacitance than the quaternary-N generated on the basal plane due to their ability to contribute in surface faradaic pseudo-capacitance in alkaline electrolytes leading to enhanced capacitance^57, 77, 78^. This pseudo-capacitance is one of the main contributors toward the observed capacitance increment in URC-900 compared with URC-1000 with lower pyridinic and pyrrolic-N in its framework. Moreover, the more appropriate distribution of micro-/mesopores in URC-900 compared with that of URC-1000 can be another reason for its larger capacitance. Therefore, URC-900 due to most favorable trade-off between key factors such as its surface area with appropriate micro-/mesopores distribution, amount of active heteroatom species, and conductivity shows the highest capacitance among all the URC electrodes. Since suitable amount of active heteroatom species and porous structure can enhance the pseudo-capacitance and electric double layer capacitance, the high specific capacitance of URC-900 is attributed to the synergistic contributions from both pseudo-capacitance and electric double layer capacitance.

**Figure 8:**
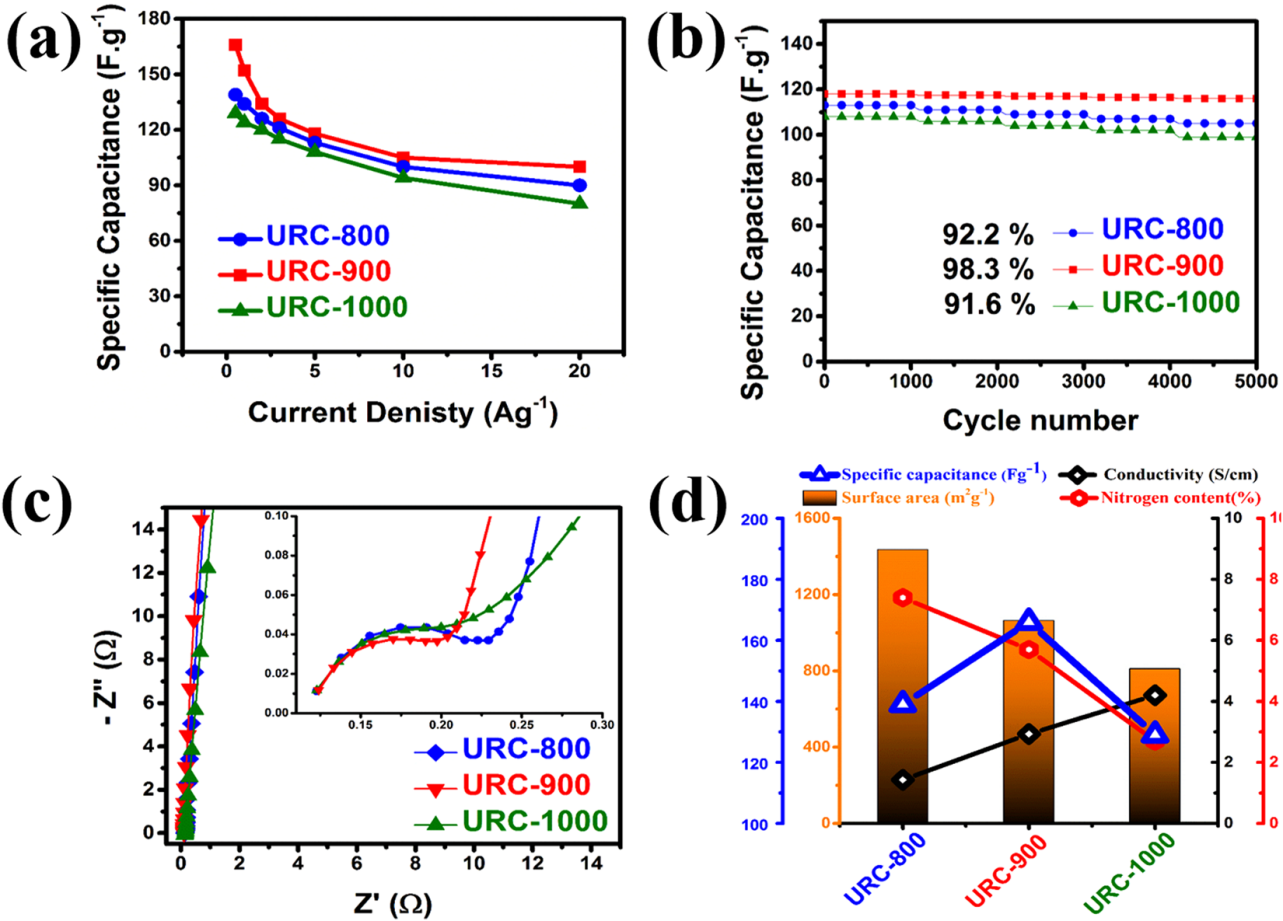
(**a**) Specific capacitance of URCs at different current densities, (**b**) cyclic stability at 5Ag^−1^ of URCs, (**c**) Nyquist impedance plots (inset: the enlarged EIS at high frequency region), and (**d**) dependence of specific capacitance on the surface area, nitrogen content, and conductivity of URCs.

Specific capacitances of all the URC samples at various current loads from 0.5 to 20 Ag^−1^ are plotted in Fig. 8a. It can be clearly seen that the capacitances of all the URC materials decay slowly with the increase of current density, suggesting a good specific capacitance retention capability of these materials even at high discharging rates, which is around 62% of their initial capacity for all the URC materials. The durability of the URCs was evaluated by the long-term charge/discharge behaviour. Figure 8b displays the capacitance of the URCs as a function of cycle number at a current density of 5 A g^−1^. The capacitance retention remains approximately within 98.3% at 5,000 cycles of charging and discharging for URC-900 compared with that of URC-800 (92.2%) and URC-1000 (91.6%), indeed indicating its long-term electrochemical stability.

Impedance spectroscopy (EIS) measurements, which provide useful information relating to electrode internal resistance and resistance between electrode and electrolyte, were conducted to study the influence of the surface area, conductivity, and heteroatom incorporation on electrochemical performances of URC electrodes. Nyquist impedance spectra in the frequency range of 10 kHz to 100 mHz at a constant potential of 0.0 V and a sinusoidal perturbation of 10 mV are shown in Fig. 8c. Z′ implies the internal resistance, whereas Z′′ shows capacitance of the capacitor. The Nyquist plot is composed of two distinguishable parts: (1) a linear and nearly vertical at low-frequency attributed to the capacitive behavior of material and diffusion process of electrolyte ions onto the surface of electrodes, and (2) a semicircle part at high frequency corresponding to the intrinsic resistance of electrodes^36^. The low-frequency part of the impedance spectrum retains almost linearity for all the URC electrodes, indicating that the electrode process is under good diffusion control^66^. The slope of these lines suggests the formation rate of the EDL^66^. It can be seen that all the samples reveal obvious half arcs at high frequency area, although they all show straight lines at lower frequency region. In the low frequency region, the slope for the URC-900 electrode is slightly larger than those of URC-800 and URC-1000, which further proves that URC-900 shows a higher capacitance and more ideal supercapacitor behavior. The semicircles part at high and medium frequency are shown in the inset of Fig. 8c. The sloped line between the semicircle and the vertical line corresponds to the pseudo-capacitive behavior of the heteroatom functionalities^40^. Presence of larger semicircle for URC-800 at high frequency is attributed to its low conductivity associated with its low carbonization temperature and to its low mesoporosity. On the other hand, URC-1000 due to its high conductivity exhibits smaller semicircle at high frequency, which is equal to lower charge transfer resistance. The results obtained by the analysis of the Nyquist plots are summarized in Table S2 of SI. As shown in Table S2 of SI, Rs values are almost similar in the range of 0.123-0.125 for all the samples since similar conditions were maintained during all the cell preparations. The interface resistance (Ri) is found to be 0.092, 0.074, and 0.086 Ω for URC-800, URC-900, and URC-1000, respectively.

To study electrolyte effect on the performance of URC-900, its supercapacitor performance has been tested in 2 M KCl as well in Fig. S11a and b of SI. As shown in Fig. S11a of SI, CV profile of URC-900 at potential scan rate of 50 mVs^−1^ displays a nearly symmetric CV curve suggesting its ideal capacitive behavior. Fig. S11b of SI shows the galvanostatic CD profile curves at various current densities for URC-900. The obtained specific capacitance is 133 F.g^−1^ for URC-900 in 2.0 M KCl, which is lower than that in 6.0 M KOH (166 F.g^−1^) at a constant current density of 0.5 Ag^−1^. The higher supercapacitor performance in KOH solution can be due to the higher ionic conductivity of KOH compared with KCl solution, which is attributed to the high mobility of OH^-^ anion in water solution and its small size^79^.

Figure 8d shows the comparison between physical, chemical, and electrochemical properties of all the obtained URC samples due to variation of surface area, nitrogen content, and conductivity as a function of carbonization temperature. Among all the URC materials, URC-900 is found to have the best performance in terms of specific capacitance. It is interesting to note that URC-800, which has a higher surface area and higher heteroatom content than those of URC-900, shows the lower capacitance than URC-900, which could be due its lower conductivity. On the other hand, although URC-1000 is highly conductive, its low capacitance might be due to the other factors such as low heteroatom content and low surface area. Based on the observation drawn from the above results, the enhanced performance of URC-900 can be addressed in terms of large surface area, and appropriate distribution of micro-/mesopores, which enable increased formation of charge double layer, along with good conductivity, high heteroatom content, and high amount of active heteroatom species, which facilitate electron and charge transfer and contribute to surface faradaic pseudo-capacitance.

In order to evaluate the supercapacitor performance of our best material, a comparative table displaying the capacitance performance of other biomass-derived renewable carbon materials and current URC-900 is shown in Table S3 of SI. It can be seen that our best sample, URC-900, generally shows better or similar capacitance performance compared to the other reported electrodes shown in earlier works prepared using different biomass precursors by different activation methods. Moreover, we have also compared the capacity retention for various biomass-derived renewable carbon materials and URC-900. It can be clearly seen that the capacity retention for the URC-900 (~98.3% at 5,000 cycles) is one of the best among all the reported ones. Therefore, we present a ‘proof of concept’ that urine, which is one of the most abundant wastes on earth and thus can cause serious environmental problems if released to open water without proper treatments, can become useful carbon electrode material with superior performance for supercapacitor without using any harmful activation or time-consuming templating process due to presence of high amount of inherent various inorganic salts, which un-necessitates the need of template or harmful porogens to create a porous framework. After all, the presence of high amount of heteroatoms and metals in the form of organic materials and salts, which can act as active sites and porogen, respectively, can make urine as an auspicious candidate to develop a new family of heteroatom-doped porous carbon materials for high performance supercapacitor without using any extra heteroatom precursor and activation agent. Therefore, using URC materials has not only a commercial advantage but also an ecological benefit.

In conclusion, highly micro-/meso porous and heteroatom-doped carbon materials with good conductivity were prepared for high performance supercapacitor by a unique, simple template-free procedure comprising of urine pyrolysis at different temperatures followed by acid-treatment for removal of inherent mineral salts. We propose that urine can be used as a single precursor for carbon and heteroatoms, while it can include a progen as well due to the presence of inherent salts embedded in its carbon framework. As heteroatom content can give rise to pseudo-capacitance and the porous structure can enhance EDL capacitance, the superior capacitance is expected from the urine-derived carbon. When tested as supercapacitor electrodes, URC-900 due to the synergistic effect of the high surface area (1040.5 m^2^g^−1^) with a proper micro-/mesopore distribution, good conductivity, and efficient heteroatom doping along with high amount of active heteroatom species doped at graphitic edges, illustrates high specific capacitance of 166 F.g^−1^ at 0.5 Ag^−1^ for three-electrode system in inorganic electrolyte among all other URC materials. Since the URC-900 has suitable amount of active heteroatom species and proper amount of micro-/mesopores in its framework, the high specific capacitance of URC-900 is attributed to the synergistic contributions from both pseudo-capacitance and electric double layer capacitance. Moreover, the URC-900 delivers outstanding cycling stability with only 1.7% capacitance decay over 5,000 cycles at 5 A g^−1^ and also delivers reasonable energy density value (12.7 to 6.10 WhKg^−1^) in the range of reasonable power density value (60.07 to 337.84 WKg^−1^). Considering the convenient and innovative template-free procedure without using any external heteroatom precursor and activation agent, and easy-availability of raw material, the current urine-based synthesis can be adjusted for scale-up industrial approach and definitely open up new dimensions in preparing new age electrodes for the application in energy fields.

## Methods

### Material synthesis

#### Synthesis of URC materials

Human urine samples with the pH around 7 to 7.5 were collected from healthy people with no prior medical examinations in high quality plastic container^47^. All the URC materials have been synthesized according to the method reported in our earlier work^47^. Briefly, after the complete carbonization of dried yellowish-brown deposit of urine obtained after its dehydration at 80 °C for 48 h, the resulted grayish black powder was removed from tube furnace and labeled as URC-X-BW, where X signifies the carbonization temperature (800, 900, and 1000 °C), and BW stands for before acid washing. After acid washing, the acid-treated carbon materials, obtained from urine carbonized at 800, 900, and 1000 °C, are labeled as URC-800, URC-900, and URC-1000, respectively.

### Material characterization

The morphology of the samples was viewed with Hitachi S-4700 SEM worked at an accelerating voltage of 10 kV and EM 912 Omega TEM operated at 120 kV. HR-TEM was performed using a Hitachi HF-3300 microscope with a field emission gun operated at 300 kV. The chemical compositions of the samples were determined by the EDX spectrometer (Hitachi HF-3300). XRD data were acquired by a Rigaku Smartlab diffractometer with Cu Kα radiation (1.5406 Å) operated at 40 kV and 40 mA. Raman spectra were obtained by a Renishaw spectrometer with an Ar^+^ ion laser (λ = 514.5 nm). XPS was conducted to examine the surface chemistry of a materials using an ESCALAB-250 spectrometer with a monochromated Al Kα (150 W) source. Micromeritics ASAP 2020 accelerated surface area and porosimetry system were used to obtain the nitrogen adsorption-desorption isotherms of all materials at −196 °C. The specific surface area was obtained based on Brunauer-Emmett-Teller (BET) method from nitrogen adsorption data at the relative pressure range between 0.05 and 0.2. Total pore volume was obtained from the amount of nitrogen adsorbed at the highest relative pressure. PSD was obtained from adsorption branch of the isotherm by the BJH method. The micropores were also characterized using H-K model. Electrical conductivity measurements for all the samples were carried out using a home-made four-point probe apparatus by varying the applied pressure according to our previous works^1, 25, 47^. The cell is made of a non-conducting Teflon block carved into a hollow cylinder covered by two metallic brass pistons, one as a base and the other as a lid, to which the pressure is applied (pressure is applied to the metallic pistons using steel plates with known weights)^80^. Carbon sample is filled in the hollow Teflon chamber. Teflon chamber was then sealed using two brass pistons, and then the pressure dependent resistivity measurements were performed by increasing the applied pressure. Voltage across the two metallic probes located in the middle of Teflon block is obtained when current is applied to the sample through the metallic pistons^80^. Keithley model 6220 with a low current noise and model 2182 A are employed as the DC current source and voltmeter, respectively. The current is altered between 0 and 10 mA and the corresponding voltage is quantified. The electrical conductivity of the samples is calculated using the equation (1)^80^:

1 $${\boldsymbol{\sigma }}={\rm{L}}/{\rm{RA}}$$

where *σ* is the electrical conductivity, *R* is the resistance of the sample, *A* is the cross sectional area of the sample (0.126 cm^2^) and *l* is the distance between the voltage probes (0.2 cm).

### Electrochemical characterization

All the URC materials were electrochemically characterized in an electrochemical workstation (VMP3, Biologic) using a conventional three-electrode system in 6.0 M aqueous KOH solution as an electrolyte, where the prepared carbon samples act as a working electrode, a coiled Pt wire as a counter electrode, and mercury/mercury oxide (Hg/HgO) as a reference electrode. 2 mm thick nickel foam (99.8% pure, MTI Corp.) was used as a current collector. In order to prepare electrode slurry, active materials (URC samples) were mixed with graphite and polyvinylidene fluoride (PVDF) in a weight ratio of 8:1:1 using a few drops of N-methyl-pyrrolidinone (NMP) as a solvent. All the mixtures were adequately stirred for 24 hr. The resulting uniform slurry was brush-coated on the nickel foam over a 1 cm^2^ area and dried overnight at room temperature followed by further overnight drying at 80 °C in a vacuum oven. The dried electrodes were then pressed at 2.0 MPa pressure to obtain a 20 mm thick electrode. Approximately, 3(±1) mg of active materials is loaded in each electrode. CV, galvanostatic CD, and EIS methods were employed for the measurement of the electrochemical performance. The CV and galvanostatic (CD) curves were recorded between −1.0 to 0.0 V at potential scan rates of 5, 10, 50, and 100 mVs^−1^ and at current densities of 0.5 to 20.0 Ag^−1^. The EIS measurements were carried out in the frequency range from 10 kHz to 100 mHz with a 10 mV AC amplitude at 0 V. The specific capacitance (C_sp_) was calculated from CD curves according to the following equation (2):

2 $${{\rm{C}}}_{{\rm{sp}}}={\rm{It}}/{\rm{m}}({{\rm{E}}}_{2}\,-\,{{\rm{E}}}_{1})$$

where I is the discharge current (A), m is the mass of the as-prepared carbon material (g), t is the time of discharge (s), and (E_2_ − E_1_) is the potential window. For two-electrode configuration, the best performance material was constructed to generate symmetric supercapacitor mode. Two electrodes were prepared with the same weight and size and used as the working and counter electrodes, and then they were symmetrically face to face integrated into the electrolyte. Two electrodes were separated by a Whatman filter paper in 6.0 M KOH electrolyte.

The energy density and power density of the electrodes at various current densities are calculated using the following equations (3) and (4):

3 $${\rm{E}}\,({{\rm{WhKg}}}^{-1})=1/2\,{\rm{C}}\,{{\rm{\Delta }}{\rm{V}}}^{2}$$

4 $${\rm{P}}\,({{\rm{WKg}}}^{-1})={\rm{E}}/{\rm{t}}$$

where E is the specific energy density, C is the specific capacitance, ΔV is the potential range, P is specific power density and t is discharging time.

## Electronic supplementary material

Supplementary Information

## References

[1] Singh KP, Song MY, Yu J-S (2014). Iodine-treated heteroatom-doped carbon: conductivity driven electrocatalytic activity. J. Mater. Chem. A.

[2] Li Z (2016). Graphene emerges as a versatile template for materials preparation. Small.

[3] Hsu H-C (2015). Graphene oxides and carbon nanotubes embedded in poly-acrylonitrile-based carbon nanofibers used as electrodes for supercapacitor. J. Phys. Chem. Solids.

[4] Béguin F (2006). Application of nanotextured carbons for electrochemical energy storage in aqueous medium. J. Braz. Chem. Soc..

[5] Zhang J (2016). Carbon science in 2016: status, challenges and perspectives. Carbon.

[6] Chen Z (2014). Graphene-based nanowire supercapacitors. Langmuir.

[7] Koo Y (2015). Inverse-ordered fabrication of free-standing CNT sheets for supercapacitor. Langmuir.

[8] Frackowiak E, Béguin F (2001). Carbon materials for the electrochemical storage of energy in capacitors. Carbon.

[9] Lv W, Li Z, Deng Y, Yang Q-H, Kang F (2016). Graphene-based materials for electrochemical energy storage devices: opportunities and challenges. Energy Storage Materials.

[10] Bhattacharjya D, Yu J-S (2014). Activated carbon made from cow dung as electrode material for electrochemical double layer capacitor. J. Power Sources.

[11] Srivastava M, Kumar M, Singh R, Agrawal U, Garg M (2009). Energy-related applications of carbon materials-a review. J. Sci. Ind. Res..

[12] Goodman PA (2013). Preparation and characterization of high surface area, high porosity carbon monoliths from pyrolyzed bovine bone and their performance as supercapacitor electrodes. Carbon.

[13] Xu Y (2015). A metal-free supercapacitor electrode material with a record high volumetric capacitance over 800 F cm^−3^. Adv. Mater..

[14] Huang H-C (2012). Pyrolyzed cobalt corrole as a potential non-precious catalyst for fuel cells. Adv. Funct. Mater..

[15] Gao Q, Demarconnay L, Piñero ER, Béguin F (2012). Exploring the large voltage range of carbon/carbon supercapacitors in aqueous lithium sulfate electrolyte. Energy Environ. Sci..

[16] Gastol D, Walkowiak J, Fic K, Frackowiak E (2016). Enhancement of the carbon electrode capacitance by brominated hydroquinones. J. Power Sources.

[17] Candelaria SL (2012). Nanostructured carbon for energy storage and conversion. Nano Energy.

[18] Xu F (2015). Facile synthesis of ultrahigh-surface-area hollow carbon nanospheres for enhanced adsorption and energy storage. Nat. Commun..

[19] Chang B, Wang Y, Pei K, Yang S, Dong X (2014). ZnCl_2_-activated porous carbon spheres with high surface area and superior mesoporous structure as an efficient supercapacitor electrode. RSC Adv..

[20] Xu B, Hou S, Cao G, Chub M, Yanga Y (2013). Easy synthesis of a high surface area, hierarchical porous carbon for high-performance supercapacitors. RSC Adv..

[21] Yang D-S, Bhattacharjya D, Song MY, Yu J-S (2014). Highly efficient metal-free phosphorus-doped platelet ordered mesoporous carbon for electrocatalytic oxygen reduction reaction. Carbon.

[22] Saha D (2014). Studies on supercapacitor electrode material from activated lignin-derived mesoporous carbon. Langmuir.

[23] Li B (2016). Nitrogen-doped activated carbon for a high energy hybrid supercapacitor. Energy Environ. Sci..

[24] Razmjooei F, Singh KP, Bae EJ, Yu J-S (2015). A new class of electroactive Fe- and P functionalized graphene for oxygen reduction. J. Mater. Chem. A.

[25] Song MY, Park H-Y, Yang D-S, Bhattacharjya D, Yu J-S (2014). Seaweed-derived heteroatom-doped highly porous carbon as an electrocatalyst for the oxygen reduction reaction. ChemSusChem..

[26] Chen L-F, Huang Z-H, Liang H-W, Gao H-L, Yu S-H (2014). Three-dimensional heteroatom-doped carbon nanofiber networks derived from bacterial cellulose for supercapacitors. Adv. Funct. Mater..

[27] Wang C-H, Hsu H-C, Hu J-H (2014). High-energy asymmetric supercapacitor based on petal-shaped MnO_2_ nanosheet and carbon nanotube-embedded polyacrylonitrile-based carbon nanofiber working at 2 V in aqueous neutral electrolyte. J. Power Sources.

[28] Chaudhari NK, Chaudhari S, Yu J-S (2014). Cube-like α-Fe_2_O_3_ supported on ordered multimodal porous carbon as high performance electrode material for supercapacitors. ChemSusChem.

[29] Miller JR, Simon P (2008). Electrochemical capacitors for energy management. Science.

[30] Faggioli E (1999). Supercapacitors for the energy management of electric vehicles. J. Power Sources.

[31] You B, Wang LL, Li N, Zheng CL (2014). Improving the energy storage performance of graphene through insertion of pristine CNTs and ordered mesoporous carbon coating. ChemElectroChem.

[32] Wen Z (2012). Crumpled nitrogen-doped graphene nanosheets with ultrahigh pore volume for high-performance supercapacitor. Adv. Mater..

[33] Qin K (2016). Free-standing 3D nanoporous duct-like and hierarchical nanoporous graphene films for micron-level flexible solid-state asymmetric supercapacitors. Adv. Energy Mater..

[34] Qin K (2016). Continuously hierarchical nanoporous graphene film for flexible solid-state supercapacitors with excellent performance. Nano Energy.

[35] Qin K (2015). Free-standing porous carbon nanofiber/ultrathin graphite hybrid for flexible solid-state supercapacitors. ACS Nano.

[36] Chen LF (2012). Synthesis of nitrogen-doped porous carbon nanofibers as an efficient electrode material for supercapacitors. ACS Nano.

[37] Zhou DD (2013). A nitrogen-doped ordered mesoporous carbon nanofiber array for supercapacitors. J. Mater. Chem. A.

[38] Jisha MR (2009). Electrochemical characterization of supercapacitors based on carbons derived from coffee shells. Mater Chem Phys..

[39] He X (2013). Efficient preparation of biomass-based mesoporous carbons for supercapacitors with both high energy density and high power density. J. Power Sources.

[40] Liang HW, Wei W, Wu ZS, Feng X, Müllen KJ (2013). Mesoporous metal-nitrogen-doped carbon electrocatalysts for highly efficient oxygen reduction reaction. J. Am. Chem. Soc..

[41] Yang D-S, Bhattacharjya D, Inamdar S, Park J, Yu J-S (2012). Phosphorus-doped ordered mesoporous carbons with different lengths as efficient metal-free electrocatalysts for oxygen reduction reaction in alkaline media. J. Am. Chem. Soc..

[42] Rufford TE, Jurcakova DH, Zhu Z, Lu GQ (2008). Nanoporous carbon electrode from waste coffee beans for high performance supercapacitors. Electrochem. Commun..

[43] Huang W, Zhang H, Huang Y, Wang W, Wei S (2011). Hierarchical porous carbon obtained from animal bone and evaluation in electric double-layer capacitors. Carbon.

[44] Kalpana D (2009). Recycled waste paper-a new source of raw material for electric double-layer capacitors. J. Power Sources.

[45] Razmjooei F, Singh KP, Song MY, Yu J-S (2014). Enhanced electrocatalytic activity due to additional phosphorous doping in nitrogen and sulfur-doped graphene: A comprehensive study. Carbon.

[46] Li Z (2013). Mesoporous nitrogen-rich carbons derived from protein for ultra-high capacity battery anodes and supercapacitors. Energy Environ. Sci..

[47] Chaudhari NK, Song MY, Yu J-S (2014). Heteroatom-doped highly porous carbon from human urine. Sci. Rept..

[48] Shrestha S, Morse N, Mustain WE (2014). Effect of surface chemistry on the double layer capacitance of polypyrrole-derived ordered mesoporous carbon. RSC Adv..

[49] Alabadi A, Yang X, Dong Z, Li Z, Tan B (2014). Nitrogen-doped activated carbons derived from a co-polymer for high supercapacitor performance. J. Mater. Chem. A.

[50] Xia K, Gao Q, Jiang J, Hu J (2008). Hierarchical porous carbons with controlled micropores and mesopores for supercapacitor electrode materials. Carbon.

[51] You B, Yin P, An L (2014). Multifunctional electroactive heteroatom-doped carbon aerogels. Small.

[52] You B, Wang L, Yao L, Yang J (2013). Three dimensional N-doped graphene–CNT networks for supercapacitor. Chem. Commun..

[53] Zhang J, Chen G, Zhang Q, Kang F, You B (2015). Self-assembly synthesis of N-doped carbon aerogels for supercapacitor and electrocatalytic oxygen reduction. ACS Appl. Mater. Interfaces.

[54] You B, Sun Y (2016). Hierarchically porous nickel sulfide multifunctional superstructures. Advanced Energy Materials.

[55] Lee WH, Moon JH (2014). Monodispersed N-doped carbon nanospheres for supercapacitor application. ACS Appl. Mater. Interfaces.

[56] Chen XY (2013). Nitrogen-doped porous carbon for supercapacitor with long-term electrochemical stability. J. Power Sources.

[57] Wang C (2013). N/P-codoped thermally reduced graphene for high-performance supercapacitor applications. J. Phys. Chem. C.

[58] Luo G (2013). Hole defects and nitrogen doping in graphene: implication for supercapacitor applications. ACS Appl. Mater. Interfaces.

[59] Jurcakova DH (2009). Nitrogen-enriched nonporous carbon electrodes with extraordinary supercapacitance. Adv. Funct. Mater..

[60] Li Y, Chen Z, Zhang J, Xu Q (2016). Dual tuning of 1 D heteroatoms doped porous carbon nanoarchitectures for supercapacitors: the role of balanced P/N doping and core@shell nanonetworks. RSC Adv..

[61] Panja T, Bhattacharjya D, Yu J-S (2015). Nitrogen and phosphorus co-doped cubic ordered mesoporous carbon as a supercapacitor electrode material with extraordinary cyclic stability. J. Mater. Chem. A.

[62] Deng W (2013). Sulfur-doped porous carbon nanosheets as an advanced electrode material for supercapacitor. RSC Adv..

[63] Zhao X (2012). Aromatic sulfide, sulfoxide, and sulfone mediated mesoporous carbon monolith for use in supercapacitor. Nano Energy.

[64] Singh K, Seredych M, Castellon ER, Bandosz TJ (2014). Effect of visible-light exposure and electrolyte oxygen content on the capacitance of sulfur-doped carbon. ChemElectroChem.

[65] Razmjooei F, Singh KP, Yu J-S (2015). Superior pore network retention of carbon derived from naturally dried ginkgo leaves and its enhanced oxygen reduction performance. Catal. Today.

[66] Hassan FM (2013). Pyrrolic-structure enriched nitrogen doped graphene for highly efficient next generation supercapacitors. J. Mater. Chem. A.

[67] Kim W (2010). Preparation of ordered mesoporous carbon nanopipes with controlled nitrogen species for application in electrical double-layer capacitors. J. Power Sources.

[68] Wang H, Maiyalagan T, Wang X (2012). Review on recent progress in nitrogen-doped graphene: synthesis, characterization, and its potential applications. ACS Catal..

[69] You B, Jiang J, Fan S (2014). Three-dimensional hierarchically porous all-carbon foams for supercapacitor. ACS Appl. Mater. Interfaces.

[70] Fang Y (2012). Renewing functionalized graphene as electrodes for high performance supercapacitors. Adv. Mater..

[71] Zhou Y (2016). Phosphorus/sulfur co-doped porous carbon with enhanced specific capacitance for supercapacitor and improved catalytic activity for oxygen reduction reaction. J. Power Sources.

[72] Juracakova DH (2009). Highly stable performance of supercapacitors from phosphorus-enriched carbons. J. Am. Chem. Soc..

[73] Fan X, Yu C, Ling Z, Yang J, Qiu J (2013). Hydrothermal synthesis of phosphate-functionalized carbon nanotube-containing carbon composites for supercapacitors with highly stable performance. ACS Appl. Mater. Interfaces.

[74] Qua J (2015). Nitrogen, oxygen and phosphorus decorated porous carbons derived from shrimp shells for supercapacitors. Electrochimica Acta.

[75] Tao Y (2013). Towards ultrahigh volumetric capacitance: graphene derived highly dense but porous carbons for supercapacitors. Sci. Rept..

[76] Senthilkumar B (2015). Highly porous graphitic carbon and Ni_2_P_2_O_7_ for a high performance aqueous hybrid supercapacitor. J. Mater. Chem. A.

[77] Ruan C, Aia K, Lu L (2014). Biomass-derived carbon materials for high-performance supercapacitor electrodes. RSC Adv..

[78] Jeong HM (2011). Nitrogen-doped graphene for high-performance ultracapacitors and the importance of nitrogen-doped sites at basal planes. Nano Lett..

[79] Wu H (2013). The effects of electrolyte on the supercapacitive performance of activated calcium carbide-derived carbon. Journal of power sources.

[80] Razmjooei F (2017). Fe-Treated Heteroatom (S/N/B/P)-Doped Graphene Electrocatalysts for Water Oxidation. ACS Catal..

