# Supplementary material for: Urine to highly porous heteroatom-doped carbons for supercapacitor: A value added journey for human waste
Source: Sci Rep. 2017 Sep 7;7:10910. doi: 10.1038/s41598-017-11229-6 (PMC5589805; doi:10.1038/s41598-017-11229-6)
Supplement: Supplementary file 1 — Supplementary Information [file 41598_2017_11229_MOESM1_ESM.doc]

Supplementary Information

**Urine to highly porous heteroatom-doped carbons for supercapacitor: A value added journey for human waste**

**Fatemeh Razmjooei ^1^ Kiranpal Singh ^1^ Tong Hyun Kang ^1^ Nitin Chaudhari,^2^ Jinliang Yuan,^3^ & Jong-Sung Yu ^1^,***

^1^Department of Energy Systems Engineering, DGIST, Daegu, 42988, Republic of Korea

^2^Department of Chemistry, Korea University, Seoul, 02841, Republic of Korea

^3^Faculty of Maritime and Transportation, Ningbo University, Ningbo 315211, China

**Corresponding Authors**

*(J.-S. Yu) E-mail: jsyu@dgist.ac.kr; Tel +82-53-785-6443.


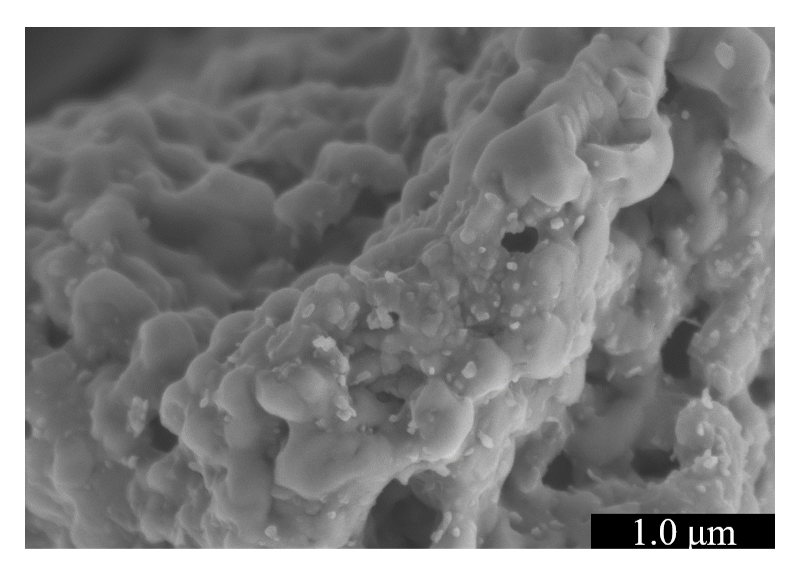


**Figure S1.** SEM image of dried yellowish-brown deposit of urine obtained at 80 ^0^C.


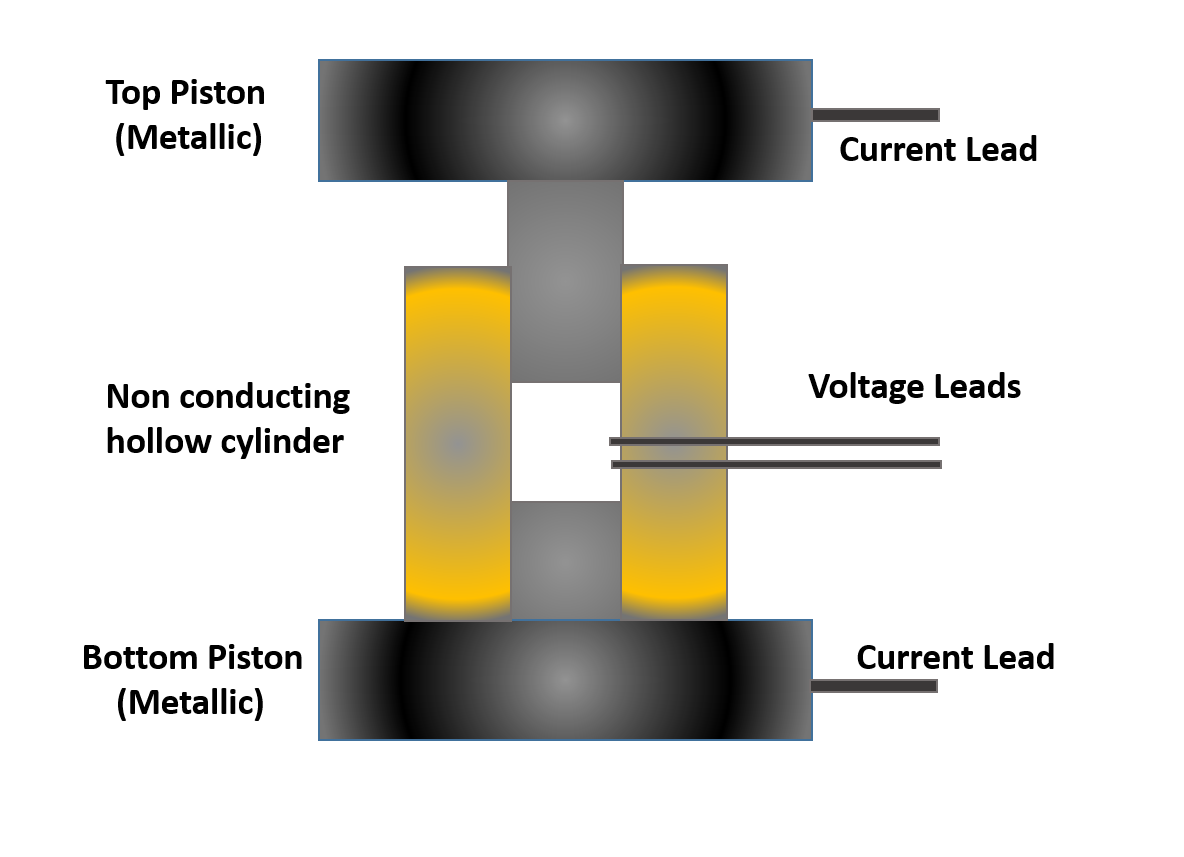


**Figure S2.** A diagram of the cell for measurement of electrical conductivity of powder samples.

**Table S1**

Physical characteristics by nitrogrn sorption data for various URC samples.

|  | Physical characteristics | | | | |
| --- | --- | --- | --- | --- | --- |
| Sample | BET total surface area (m^2^g^-1^) | Micropore surface area (m^2^g^-1^) | Pore volume (cm^3^g^-1^) | Micropore volume  (cm^3^g^-1^) | Pore size by BJH (nm) |
| URC-800 | 1330.6 | 1290.7 | 0.82 | 0.72 | 2.8 |
| URC-900 | 1040.5 | 895.2 | 0.65 | 0.45 | 3.0 |
| URC-1000 | 809.3 | 226.5 | 0.57 | 0.14 | 3.1 |


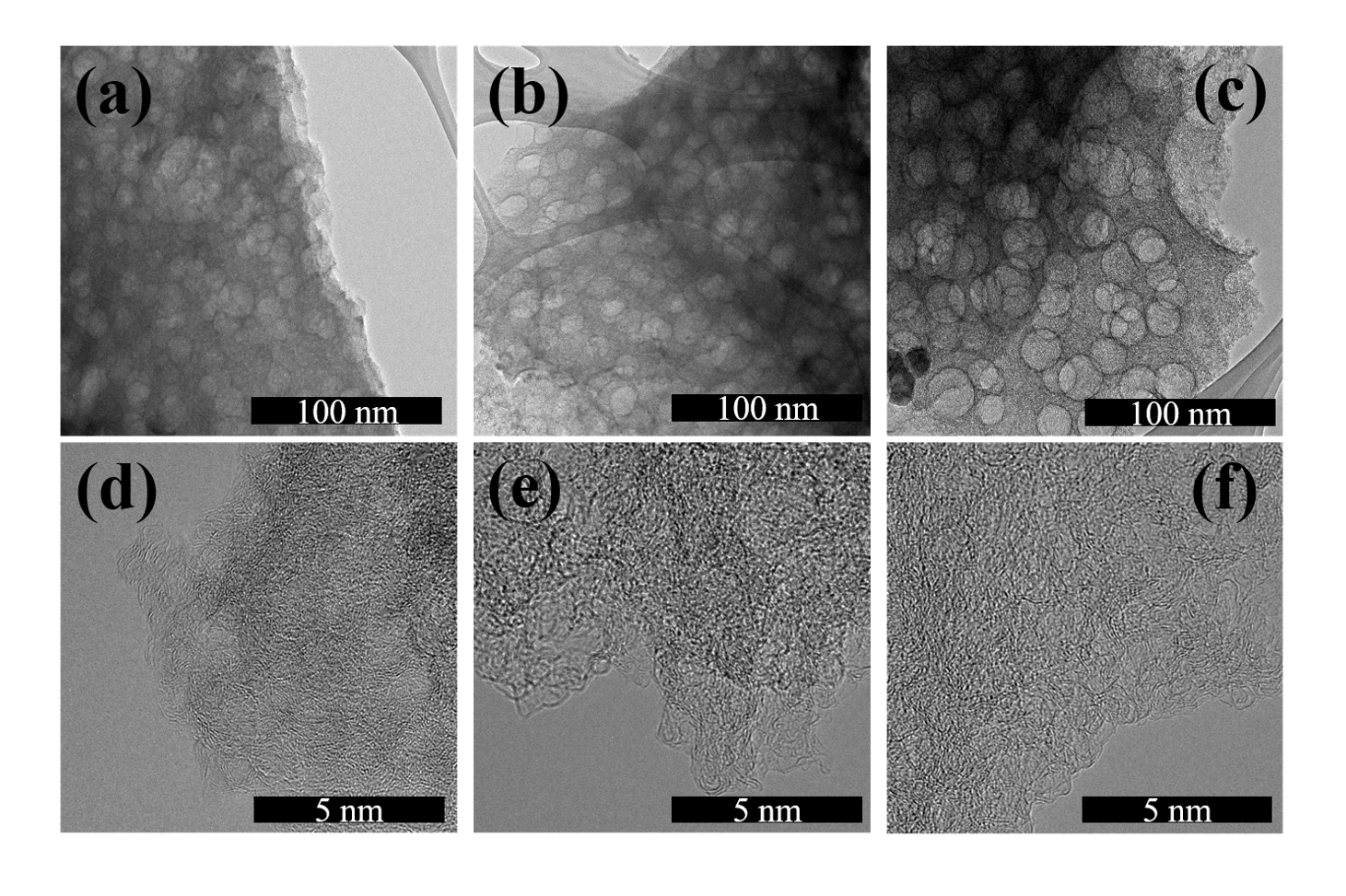


**Figure S3**. Low and high magnification HR-TEM images of URC materials: (a,d) URC-800, (b,e) URC-900, and (c,f) URC-1000.


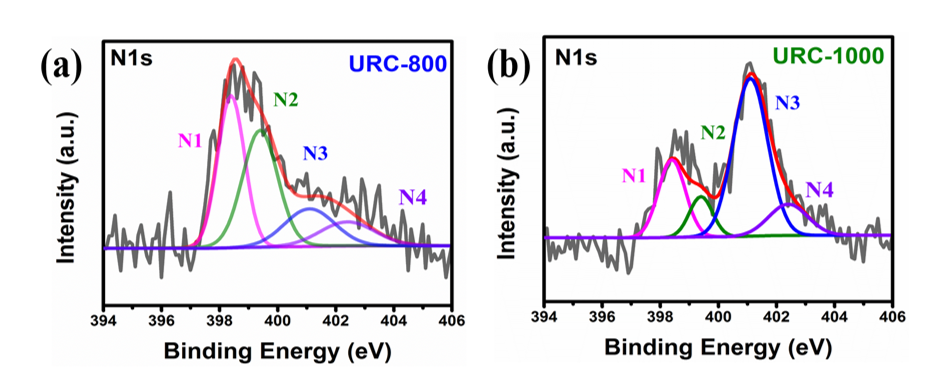


**Figure S4.** Deconvoluted XPS spectra of N 1s for (a) URC-800 and (b) URC-1000.


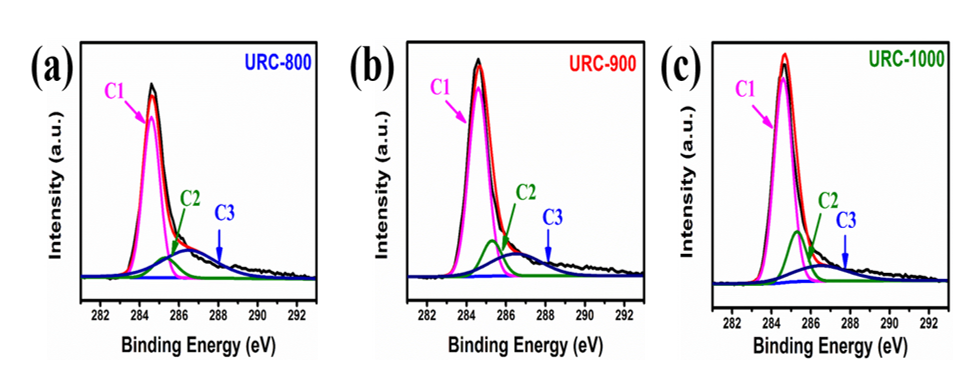


**Figure S5.** Deconvoluted XPS spectra of C 1s for (a) URC-800, (b) URC-900, and (c) URC-1000.


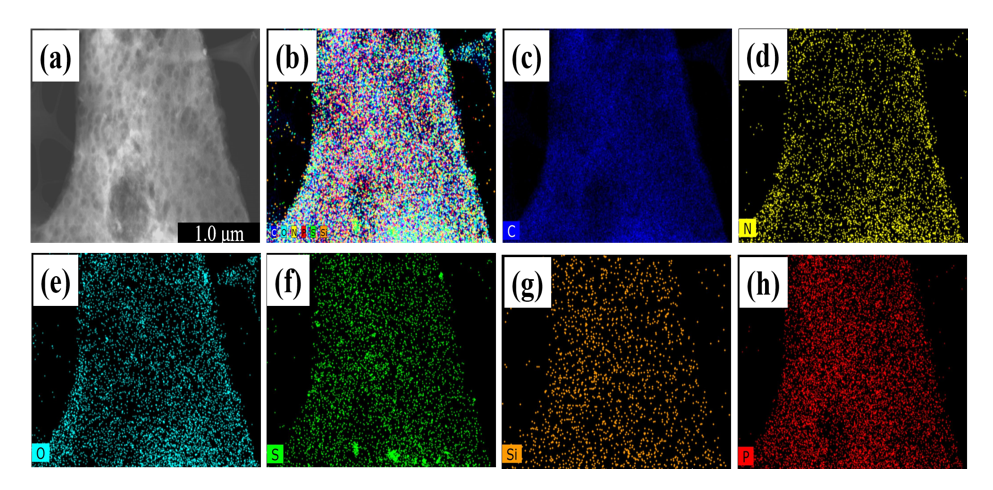


**Figure S6.** EDX elemental mapping analysis of URC-900. (a,b) Selected area and corresponding elemental mappings for (c) C, (d) N, (e) O, (f) S, (g) Si, and (h) P.


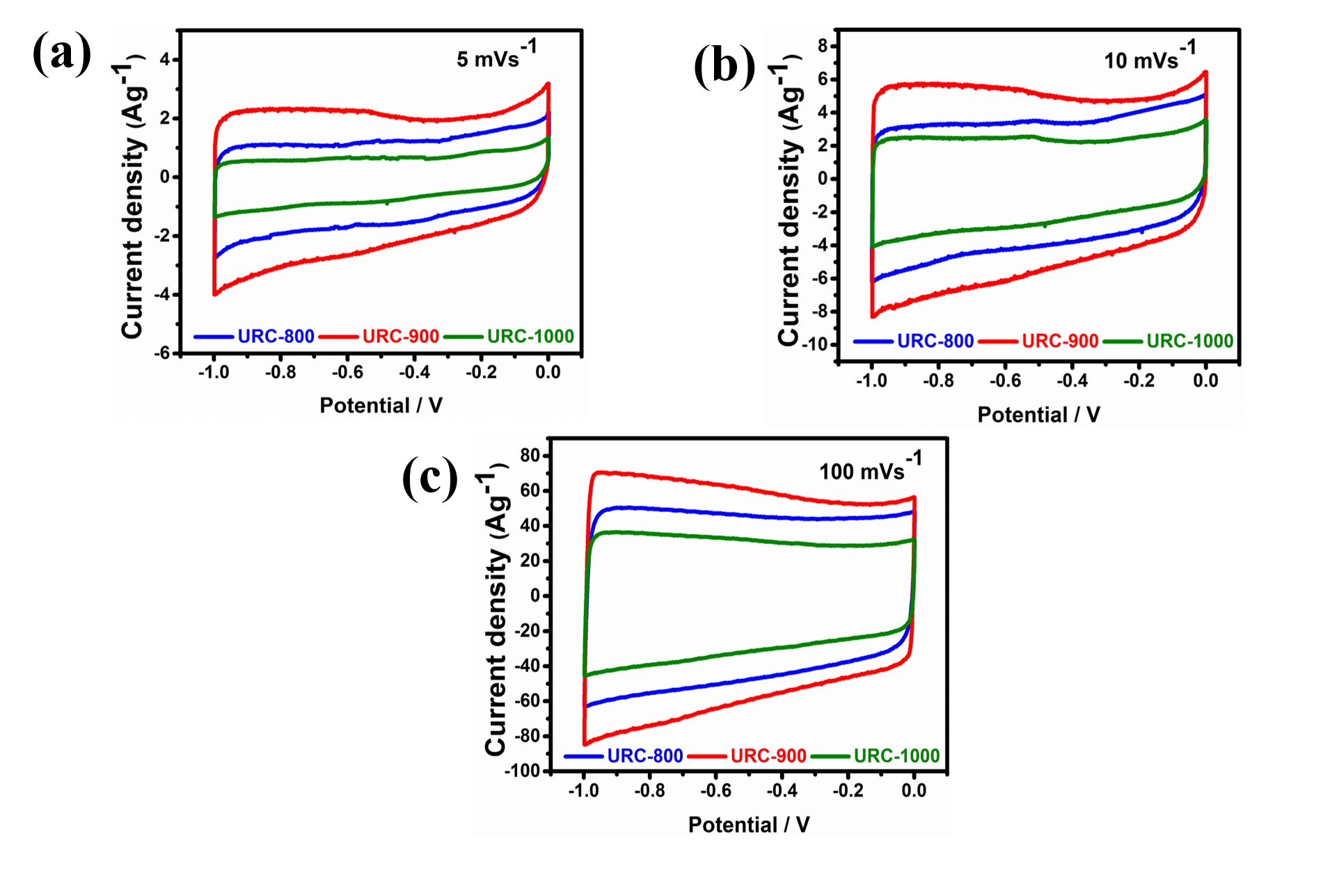


**Figure S7.** CVs of URC materials at (a) 5, (b) 10, and (c) 100 mV s^-1^ potential scan rates.


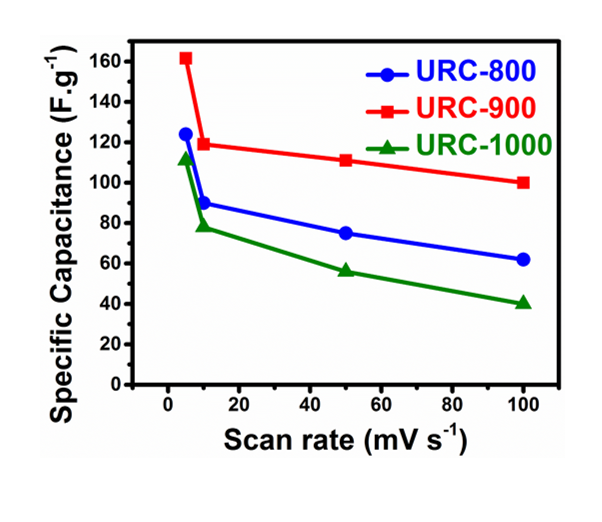


**Figure S8**. Specific capacitance of URCs at different scan rates obtained from CV.


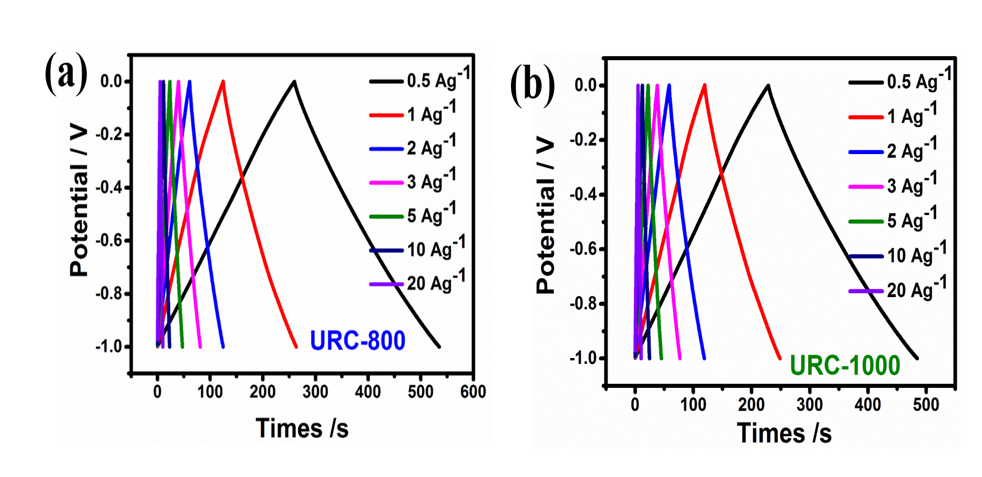
 **Figure S9.** Galvanostatic CD profiles of (a) URC-800 and (b) URC-1000 at different current densities from 0.5 to 20 A g^-1^.


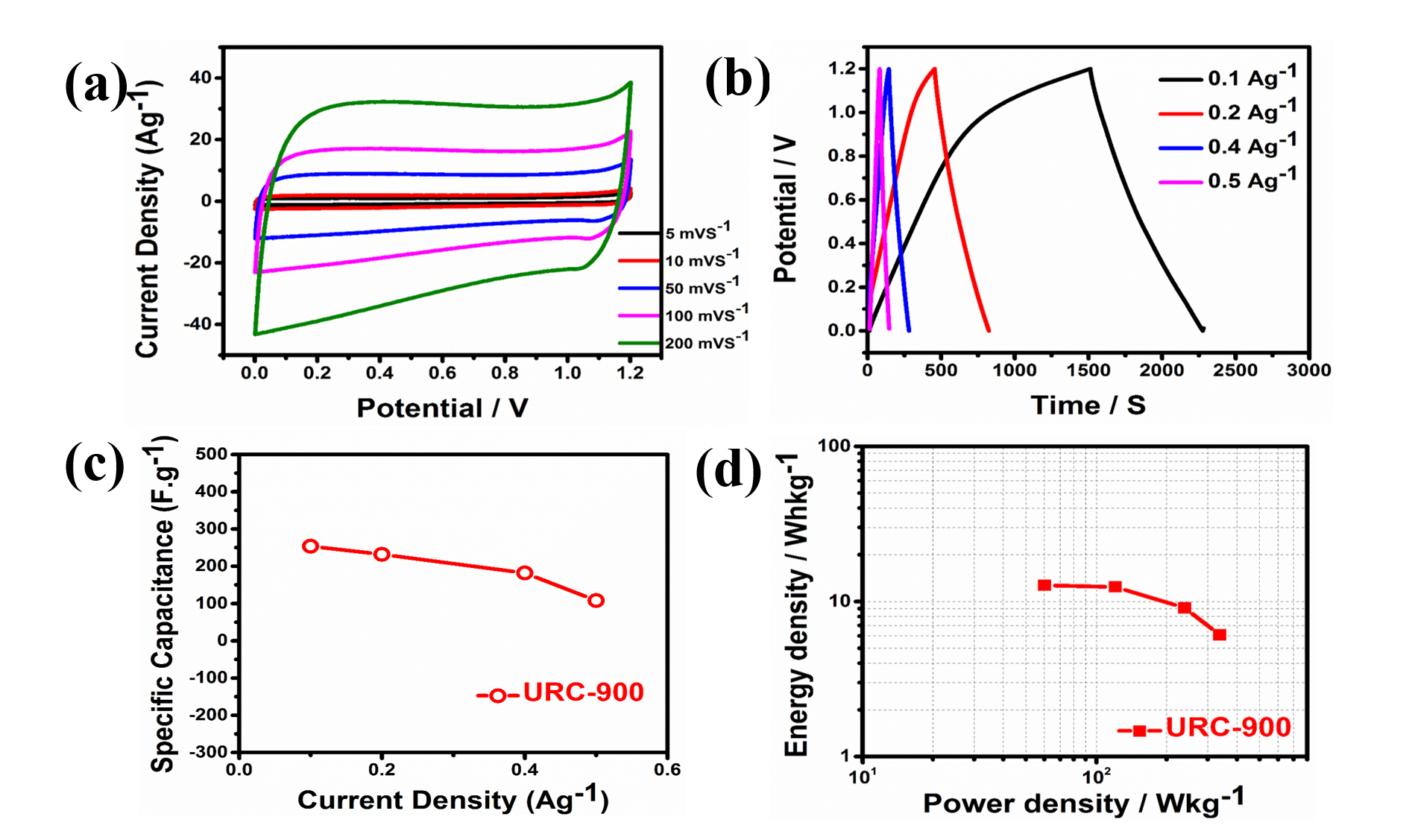


**Figure S10.** Electrochemical performance of obtained from two-electrode system. (a) CV curves in 6.0 M KOH at different scan rates, (b) galvanostatic charge/discharge curves at different current densities, (c) specific capacitances under different current densities, and (d) Ragone plot for URC-900.


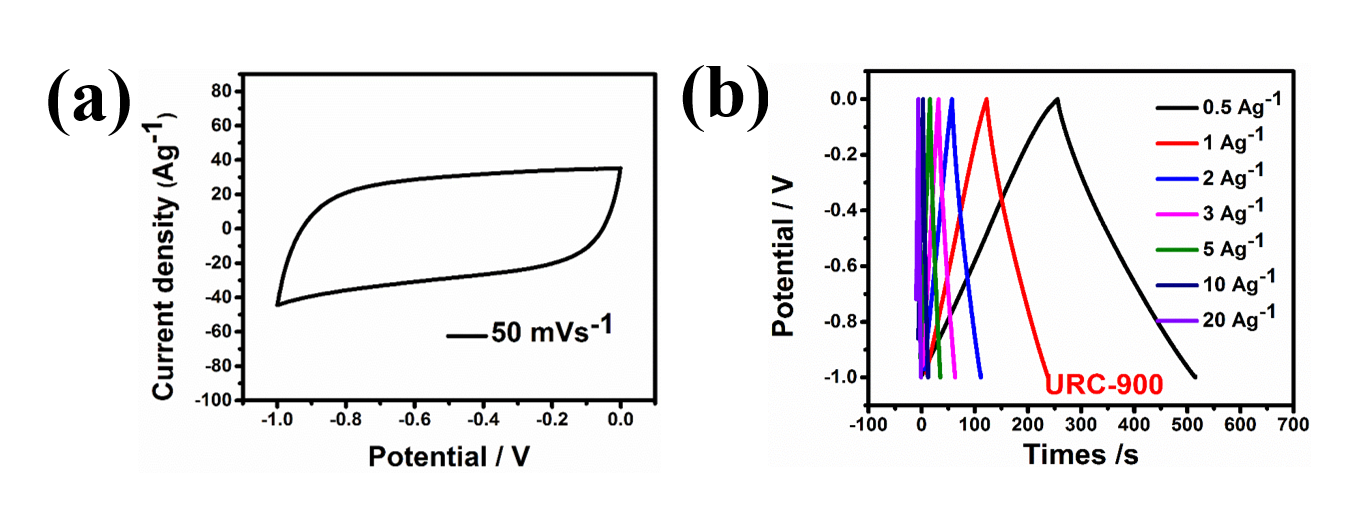


**Figure S11.** (a) CV profile of URC-900 at potential scan rate of 50 mVs^-1^ and (b) galvanostatic CD profiles of URC-900 at different current densities from 0.5 to 20 Ag^-1^ in 2M KCl.

**Table S2**

**EIS parameters of URCs obtained from analysis of Nyquist plot.**

| Sample | R_s_ (Ω) | R_i_ (Ω) | W(ΩS^-1/2^) |
| --- | --- | --- | --- |
| URC-800 | 0.123 | 0.097 | 0.035 |
| URC-900 | 0.125 | 0.085 | 0.031 |
| URC-1000 | 0.124 | 0.075 | 0.028 |

**Table S3**

Comparison of electrochemical properties of URC-900 and various biomass-derived renewable carbon materials for supercapacitor.

| **Precursor** | **Activation method** | **Surface area**  **BET-SSA**  **(m2 g^−1^)** | **Specific capacitance (F/g)** | **cycling stability** | **Ref.** |
| --- | --- | --- | --- | --- | --- |
| Urine | --- | 1040.5 | 166 | 98.3% at 5,000 cycles | **This work** |
| Banana fibers | ZnCl_2_ | 1097 | 74 | 88% at 5,000 cycles | S1 |
| Banana fibers | KOH | 686 | 66 | 70% at 5,000 cycles | S1 |
| Fire wood | H_2_O | 1131 | 114 | --- | S2 |
| Fire wood | H_2_O | 1131 | 142 | --- | S2 |
| Fire wood | H_2_O | 1131 | 142 | --- | S2 |
| Fish scale | Pyrolysis | 2273 | 168 | --- | S3 |
| Cassava peel | KOH & CO_2_ | 1325 | 153 | --- | S4 |
| Pistachio shell | KOH & CO_2_ | 1013 | 122 | 77% at 200 cycles | S5 |
| Pistachio shell | KOH | 1013 | 81 | --- | S5 |
| Sunflower seed shell | KOH | 1235 | 144 | --- | S6 |
| Coffee shells | ZnCl_2_ | 842 | 158 | --- | S7 |
| Coconut kernel | KOH | 1200 | 173 | --- | S8 |
| Water hyacinth | KOH/microwave assisted | 1010 | 179.6 | 94% at 1000 cycles | S9 |
| Animal bones | HNO_3_ | 2157 | 130 | --- | S10 |
| Feathers | KOH | 1839 | 168 | 83% at 5000 cycles | S11 |

**References**

S1. Subramanian, V.; Luo, C.; Stephan, A. M.; Nahm, K. S.; Thomas, S. & Wei, B. Supercapacitors from activated carbon derived from banana fibers. *Journal of Physical Chemistry C* **111**, 7527-7531 (2007).

S2. Wu, F. C.; Tseng, R. L.; Hu, C. C. & Wang, C. C. Physical and electrochemical characterization of activated carbons prepared from firwoods for supercapacitors. *J. Power Sources* **138**, 351-359 (2004).

S3. Chen, W. X.; Zhang, H.; Huang, Y. Q. & Wang, W. K. Fish scale based hierarchical lamellar porous carbons obtained by natural template for high performance electrochemical capacitors. *J. Mater. Chem*. **20**, 4773-4775 (2010).

S4. Ismanto, A. E.; Wang, S.; Soetaredjo, F. E. & Ismadji, S. Preparation of capacitor’s electrode from cassava peel waste, *Bioresour. Tech*. **101**, 3534-3540 (2010).

S5. Hu, C. C.; Wang C. C.; Wu, F. C. & Tseng, R. L. Characterization of pistachio shell-derived carbons activated by a combination of KOH and CO_2_ for electric double-layer capacitors. *Electrochim. Acta* **52**, 2498-2505 (2007).

S6. Li, X.; Xing, W.; Zhuo, S.; Zhou, J.; Li, F.; Qiao, S. Z. & Lu, G. Q. Preparation of capacitor’s electrode from sunflower seed shell. *Bioresource technology* **102**, 1118-1123 (2011).

S7. Jisha, M. R.; Wang, Y. J. H.; Shin, J. S.; Nahm, K. S.; Kumar, T. P.; Karthikeyan, K.; Dhanikaivelu, N.; Kalpana, D.; Renganathan, N. G. & Stephan, A. M. Electrochemical characterization of supercapacitors based on carbons derived from coffee shells. *Materials Chemistry and Physics* **115**, 33-39 (2009).

S8. Wang, R.; Wang, P.; Yan, X.; Lang, J.; Peng, C. & Xue, Q. Promising porous carbon derived from celtuce leaves with outstanding supercapacitance and CO_2_ capture performance. *ACS Applied Materials & Interfaces* **4**, 5800-5806 (2012).

S9. Kurniawan, F.; Wongso, M.; Ayucitra, A.; [Soetaredjo](https://www.researchgate.net/profile/Felycia_Edi-Soetaredjo), F. E.; Angkawijaya, A. E.; Ju, Y. H. & Ismadji, S. Carbon microsphere from water hyacinth for supercapacitor electrode. *Journal of the Taiwan Institute of Chemical Engineers* **47**, 197-201 (2015).

S10. W. Huang, H. Zhang, Y. Huang, W. Wang & S. Wei. Hierarchical porous carbon obtained from animal bone and evaluation in electric double-layer capacitors. *Carbon* **49**, 838-843 (2011).

S11. Wang, Q.; Cao, Q.; Wang, X.; Jing, B.; Kuang, H. & Zhou, L. A high-capacity carbon prepared from renewable chicken feather biopolymer for supercapacitors. *Journal of Power Sources* **225**, 101-107 (2013).
